# State COVID-19 Vaccine Mandates and Uptake Among Health Care Workers in the US

**DOI:** 10.1001/jamanetworkopen.2024.26847

**Published:** 2024-08-14

**Authors:** Yin Wang, Charles Stoecker, Kevin Callison, Julie H. Hernandez

**Affiliations:** 1 Department of Health Policy and Management, School of Public Health and Tropical Medicine, Tulane University, New Orleans, Louisiana; 2 Department of International Health and Sustainable Development, School of Public Health and Tropical Medicine, Tulane University, New Orleans, Louisiana

## Abstract

**Importance:**

Seventeen states introduced COVID-19 vaccine mandates for health care workers (HCWs) in mid-2021. Prior research on the effect of these mandates was centered on the nursing home sector, and more evidence is needed for their effect on the entire HCW population.

**Objective:**

To examine the association between state COVID-19 vaccine mandates for HCWs and vaccine uptake in this population.

**Design, Setting, and Participants:**

This repeated cross-sectional study included biweekly, individual-level data for adults aged 25 to 64 years who were working or volunteering in health care settings obtained from the Household Pulse Survey between May 26 and October 11, 2021. Analyses were conducted between November 2022 and October 2023.

**Exposure:**

Announcement of a state COVID-19 vaccine mandate for HCWs.

**Main Outcomes and Measures:**

An indicator for whether a sampled HCW ever received a COVID-19 vaccine and an indicator for whether an HCW completed or intended to complete the primary COVID-19 vaccination series. Event study analyses using staggered difference-in-differences methods compared vaccine uptake among HCWs in mandate and nonmandate states before and after each mandate announcement. The sample was further stratified by the availability of regular COVID-19 testing in place of a vaccination (ie, a test-out option) and by the ages of HCWs (25-49 or 50-64 years) to examine heterogeneous associations.

**Results:**

The study sample included 31 142 HCWs (mean [SD] age, 45.5 [10.6] years; 72.1% female) from 45 states, 16 of which introduced COVID-19 vaccine mandates for HCWs. Results indicated a mandate-associated 3.46–percentage point (pp) (95% CI, 0.29-6.63 pp; *P* = .03) increase in the proportion of HCWs ever vaccinated against COVID-19 and a 3.64-pp (95% CI, 0.72-6.57 pp; *P* = .02) increase in the proportion that completed or intended to complete the primary vaccination series 2 weeks after mandate announcement from baseline proportions of 87.98% and 86.12%, respectively. In the stratified analyses, positive associations were only detected in mandate states with no test-out option and among HCWs aged 25 to 49 years, which suggested vaccination increases of 3.32% to 7.09% compared with baseline proportions.

**Conclusions and Relevance:**

This repeated cross-sectional study found that state COVID-19 vaccine mandates for HCWs were associated with increased vaccine uptake among HCWs, especially among younger HCWs and those in states with no test-out option. These findings suggest the potential for vaccine mandates to further promote vaccinations in an already highly vaccinated HCW population, especially when no test-out option is in place.

## Key Points

**Question:**

Were state COVID-19 vaccine mandates for US health care workers (HCWs) associated with increased vaccine uptake in this population in 2021?

**Findings:**

In this cross-sectional study of 31 142 HCWs sampled across the US, state COVID-19 vaccine mandates for HCWs were associated with increases in the proportions of ever vaccinated HCWs and those who completed or intended to complete the vaccination series 2 weeks after mandate announcement relative to baseline proportions of 88% and 86%, respectively.

**Meaning:**

These findings suggest that state COVID-19 vaccine mandates were associated with increased vaccine uptake among HCWs in 2021.

## Teaser

This cross-sectional study explores associations between state-imposed COVID-19 vaccination mandates and vaccine uptake among health care workers in 2021.

## Introduction

Since their debut in 1796, vaccines have played a pivotal role in controlling lethal epidemics, such as smallpox, cholera, and bubonic plague.^1^ Yet, the decision to vaccinate relies not only on the efficacy of vaccines but also on complex factors involving personal experience, religious beliefs, and societal attitudes,^2^ ultimately driving the need for policy interventions to support widespread immunization. Among them, vaccine mandates, first introduced to combat smallpox outbreaks in the early 19th century,^3^ have remained a critical public health strategy, especially within the sensitive environment of health care settings.

Before the COVID-19 pandemic, more than 20 states in the US had introduced influenza vaccine requirements for health care workers (HCWs) in long-term care and/or hospital settings, and more than a dozen adopted similar requirements for hepatitis B and measles, mumps, and rubella immunization.^4^ These practices laid groundwork for similar interventions during the COVID-19 pandemic. In mid-2021, in response to the increasing number of new cases of the Delta variant, 17 states introduced COVID-19 vaccine mandates for HCWs in multiple settings (eg, hospitals, ambulatory care facilities, and nursing homes) after measures of social distancing, mask mandates, and vaccination incentives had been applied for the general population (eTable 1 in Supplement 1). Mississippi first announced a nursing home employee mandate on June 14, 2021, followed by California and New York with mandates for all HCWs in late July and then 14 other states in August. All these mandates were implemented with medical and/or religious exemptions. Six mandate states also allowed for regular COVID-19 testing in place of vaccination (ie, a test-out option). At the federal level, in accordance with the Biden Administration’s COVID-19 response plan, the Centers for Medicare & Medicaid Services (CMS) announced its intention to mandate COVID-19 vaccination for employees of nursing homes receiving federal funding on August 18, 2021,^5^ and then restated its intention of mandating staff vaccination in all funded health care facilities on September 9, 2021,^6^ with the final rule on implementation details officially released on November 4, 2021.^7^

Intensive debate ensued concerning health care ethics, personal autonomy, and public welfare tradeoffs.^8,9,10^ Divergent attitudes were also found among HCWs, with acceptance rates for vaccine mandates varying from 35% to 92% depending on different sampled cohorts.^11^ These findings warrant more empirical evidence on the effect of these vaccine mandates. To our knowledge, however, among the published research analyzing the impact of COVID-19 vaccine mandates in the US, only 2 studies focused on HCW mandates (specifically, nursing home worker mandates),^12,13^ with another 3 examining non-HCW mandates for municipal employees^14^ or college students^15^ or vaccination requirements for entering public venues (vaccine passports).^16^ As far as we are aware, a study on the effect of COVID-19 vaccine mandates on the entire HCW population has yet to be conducted. In this repeated cross-sectional study, we added to the current literature by using nationally representative data from the Household Pulse Survey (HPS) to explore the association between state COVID-19 vaccine mandates for HCWs and changes in vaccine uptake in this population. We further explored whether the association differed by the stringency of mandates (ie, availability of a test-out option) and the ages of HCWs.

## Methods

### Data

Of the 17 states that introduced COVID-19 vaccine mandates for HCWs in mid-2021, we included 16 states in the mandate group (California, Colorado, Connecticut, Delaware, Illinois, Maine, Maryland, Massachusetts, New Jersey, New Mexico, New York, Oregon, Pennsylvania, Rhode Island, Washington, and Washington, DC) and excluded Mississippi, as its mandate was announced more than a month earlier than other states and was only in effect until September 30, 2021. We also excluded 5 states with HCW mandates that were rescinded shortly after enactment or with other mandates that may have affected HCWs and included 29 states in the control group (Alabama, Alaska, Arizona, Arkansas, Florida, Georgia, Indiana, Iowa, Kansas, Louisiana, Michigan, Missouri, Montana, Nebraska, New Hampshire, North Carolina, North Dakota, Ohio, Oklahoma, South Carolina, South Dakota, Tennessee, Texas, Utah, Vermont, Virginia, West Virginia, Wisconsin, and Wyoming) (details in eAppendix 1 in Supplement 1). The Tulane University institutional review board exempted the study from approval and informed consent because only publicly available, deidentified data were used. We followed the Strengthening the Reporting of Observational Studies in Epidemiology (STROBE) reporting guideline for time-dependent estimates in cross-sectional analyses. We obtained biweekly, individual-level data from the HPS, initiated by the US Census Bureau in April 2020 to collect information on how individuals and households have been affected by critical social and economic matters since the COVID-19 pandemic.^17^ Although the HPS is an experimental data product,^18^ it provides valuable data with a rapid turnaround time^17^ and has facilitated multiple studies on policy evaluation and other topics.^19,20,21^ We restricted the sample to working-age adults between 25 and 64 years of age who were working or volunteering in health care settings based on response to the question, “Since January 1, 2021, which best describes the primary location/setting where you worked or volunteered outside your home?” Following prior research on vaccine mandates,^13,14^ we considered the timing of the mandate announcement as the start of the intervention, as HCWs were required to complete the requisite vaccine doses before the mandate’s enforcement date. The study window encompassed survey weeks 31 to 39, from May 26 (2 months before the earliest mandate announcement in states with mandates) to October 11, 2021. We excluded later survey waves as they extended into release of the nationwide HCW mandate by the CMS on November 4, 2021, which may have contaminated our results for state HCW mandates. A survey week spanned 2 calendar weeks. The studied mandates were announced during survey weeks 34 to 36.

### Outcome Measures

We generated 2 binary outcome variables: COVID-19 vaccine uptake status and COVID-19 vaccine primary series completion status. The former was based on the survey question “Have you received a COVID-19 vaccine?” The latter was based on the question “Did you receive (or do you plan to receive) all required doses?” The analysis also controlled for self-reported individual characteristics, including sex, age, race (Black, White, and other [included Asian, multiracial, and any other race, which were combined into a single category because of small sample sizes]), ethnicity (Hispanic, non-Hispanic), marital status, educational attainment, and income levels, and the lagged intensity of the COVID-19 pandemic (measured by COVID-19 death counts in each state within the 2 weeks before each survey week divided by the state’s population in 2021). Race and ethnicity were included as covariates to account for heterogeneous vaccination behaviors and access to health care resources based on race and ethnicity.

### Statistical Analysis

We used difference-in-differences (DID) event study models to explore the dynamic policy impact in periods prior to and following the announcement of state COVID-19 vaccine mandates for HCWs. The validity of the DID design depended on whether states with and without mandates would have trended similarly in the absence of mandate enactment (ie, the parallel-trends assumption). While this assumption is not directly testable, our event study model provided lead estimates relative to the baseline period that could be used to assess divergent preperiod trends and lag estimates that illustrated the policy impact in each period after mandate announcement.

Specifically, we used a staggered DID method proposed by Sun and Abraham^22^ that was built on the event study design. Compared with the conventional 2-way fixed effects (TWFE) model, this method accommodates staggered policy adoptions and impact of heterogeneous treatment across treated units, which was appropriate in our study given the timing of mandate enactment and the potential for state vaccination rate trajectories to differ with time since mandate announcement. An in-depth description is available in eAppendix 2 in Supplement 1, where we also provided results obtained using the TWFE estimator and another staggered DID estimator proposed by Callaway and Sant’Anna^23^ as a robustness test for different estimation methods. We also incorporated individual-level survey weights, which denote the inverse probability of sampling an individual into the HPS, in our analysis and clustered SEs at the state level to account for serial correlation in vaccine uptake within a state over time. Analyses were conducted using Stata, version 16 (StataCorp LLC). Statistical tests were 2-sided, with *P* < .05 considered statistically significant. Analyses were conducted between November 2022 and October 2023.

## Results

The sample included 31 142 HCWs (mean [SD] age, 45.5 [10.6] years; 24 294 [72.1%] female; 6848 [27.9%] male). A total of 2927 (15.3%) were Black, 24 951 (71.7%) were White, and 3264 (13.0%) were other race; 2555 (12.8%) were Hispanic, and 28 587 (87.2%) were non-Hispanic. Of the total HCWs, 12 431 (43.4%) were from mandate states, and 18 711 (56.6%) were from nonmandate states (Table). Compared with HCWs in control states, HCWs in states with mandates were more likely to be Hispanic (1326 [15.8%] vs 1229 [10.5%]) and college graduates (8262 [50.5%] vs 11 881 [47.4%]) and to have an annual household income of $100 000 or more (5353 [35.4%] vs 7086 [29.9%]); they were less likely to be married (7379 [58.3%] vs 11 918 [62.6%]), Black (1210 [14.3%] vs 1717 [16.1%]), or White (9401 [66.5%] vs 15 550 [75.7%]). A large proportion of sampled HCWs had been vaccinated in the premandate period (12 172 [84.2%]), with a higher proportion of HCWs in mandate states (5147 [87.5%]) reporting premandate vaccination than HCWs in control states (7025 [81.6%]).

**Table.** Descriptive Statistics of Sampled Health Care Workers

Characteristic	Health care workers, unweighted No. (weighted %)^a^		
	All (N = 31 142)	Mandate states (n = 12 431)^b^	Nonmandate states (n = 18 711)^c^
Sex			
Female	24 294 (72.1)	9554 (71.2)	14 740 (72.8)
Male	6848 (27.9)	2877 (28.8)	3971 (27.2)
Marital status			
Unmarried	11 845 (39.3)	5052 (41.7)	6793 (37.4)
Married	19 297 (60.7)	7379 (58.3)	11 918 (62.6)
Race			
Black	2927 (15.3)	1210 (14.3)	1717 (16.1)
White	24 951 (71.7)	9401 (66.5)	15 550 (75.7)
Other^d^	3264 (13.0)	1820 (19.2)	1444 (8.2)
Ethnicity			
Hispanic	2555 (12.8)	1326 (15.8)	1229 (10.5)
Non-Hispanic	28 587 (87.2)	11 105 (84.2)	17 482 (89.5)
Age, y			
25-34	5700 (28.6)	2300 (29.0)	3400 (28.4)
35-49	13 547 (39.3)	5327 (38.5)	8220 (39.9)
50-64	11 895 (32.0)	4804 (32.5)	7091 (31.7)
Educational level			
Less than college degree	10 999 (51.3)	4169 (49.5)	6830 (52.6)
College degree or higher	20 143 (48.7)	8262 (50.5)	11 881 (47.4)
Annual household income, $			
<35 000	2831 (12.4)	1020 (11.1)	1811 (13.4)
35 000-100 000	9778 (32.0)	3583 (29.4)	6195 (34.0)
>100 000	12 439 (32.3)	5353 (35.4)	7086 (29.9)
Income unknown	6094 (23.3)	2475 (24.0)	3619 (22.7)
Vaccine uptake outcomes			
Ever vaccinated in premandate period^e^	12 172 (84.2)	5147 (87.5)	7025 (81.6)
Ever vaccinated in postmandate period^f^	16 038 (89.9)	6449 (93.4)	9589 (87.2)
Primary series completed in premandate period^g^	11 978 (82.5)	5067 (86.1)	6911 (79.7)
Primary series completed in postmandate period	15 944 (89.3)	6411 (92.8)	9533 (86.7)

^a^ Percentage estimates were weighted using survey weights.

^b^ California, Colorado, Connecticut, Delaware, Illinois, Maine, Maryland, Massachusetts, New Jersey, New Mexico, New York, Oregon, Pennsylvania, Rhode Island, Washington, and Washington, DC.

^c^ Alabama, Alaska, Arizona, Arkansas, Florida, Georgia, Indiana, Iowa, Kansas, Louisiana, Michigan, Missouri, Montana, Nebraska, New Hampshire, North Carolina, North Dakota, Ohio, Oklahoma, South Carolina, South Dakota, Tennessee, Texas, Utah, Vermont, Virginia, West Virginia, Wisconsin, and Wyoming.

^d^ The Household Pulse Survey categorizes race as Asian, Black, White, multiracial, and any other race. This study grouped Asian, multiracial, and any other race as “other” because of small sample sizes in these categories.

^e^ For comparison purposes, the premandate period included survey weeks 31 to 33, when no mandates had been announced in states that later instituted them.

^f^ For comparison purposes, the postmandate period included survey weeks 34 to 39, from when mandates were gradually initiated in mandate states.

^g^ Health care workers who completed or intended to complete the primary series of COVID-19 vaccination.

**Figure 1.**
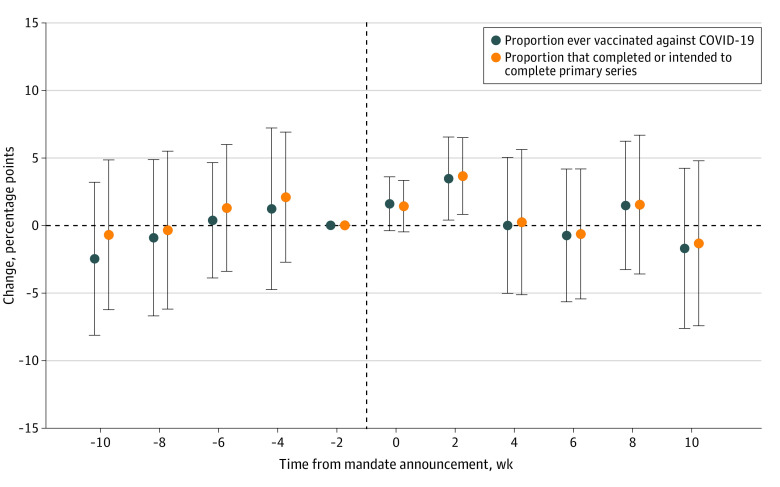
Event Study of the Association Between State COVID-19 Vaccine Mandates and Uptake Among Health Care Workers in the Full Sample The plotted points are regression coefficients on a series of indicators for survey weeks before and after the announcement of state vaccine mandates, derived from a staggered difference-in-differences analysis described in the Methods section. The vertical dashed line represents the mandate announcement. All estimates are relative to the survey week immediately prior to the mandate announcement and were weighted via survey weights. A survey week spanned 2 calendar weeks. Error bars indicate 95% CIs. Models controlled for state fixed effects, survey week fixed effects, individual characteristics (sex, age, race, ethnicity, marital status, educational level, and income level), and the lagged intensity of the COVID-19 pandemic of each state. Standard errors were clustered at the state level. Additional details are given in eAppendix 3 in Supplement 1.

Figure 1 displays event study estimates of the change in the vaccinated proportion of HCWs in mandate states compared with nonmandate states in each period before and after mandate announcement (additional details in eAppendix 3 in Supplement 1). The survey week immediately prior to the mandate announcement was used as the baseline to facilitate easy comparisons between the postmandate period and the rest of the premandate period. In the postmandate period, statistically significant increases in vaccine uptake were found 2 weeks after mandate announcement, but these dissipated in the following periods. Specifically, the mandates were associated with an increase of 3.46 percentage points (pp) (95% CI, 0.29-6.63 pp; *P* = .03) in the proportion of HCWs ever vaccinated against COVID-19 in mandate states compared with nonmandate states 2 weeks after mandate announcement, representing a 3.93% increase relative to a baseline proportion of 87.98%. A slightly larger increase (3.64 pp; 95% CI, 0.72-6.57 pp; *P* = .02) was found for the proportion of HCWs who completed or intended to complete the primary vaccine series in the same period, representing a 4.23% increase relative to a baseline proportion of 86.12%. Estimates unadjusted for covariates and by Callaway and Sant’Anna^23^ and TWFE estimators revealed similar findings (eFigures 1-4 and eTables 2 and 3 in Supplement 1).

**Figure 2.**
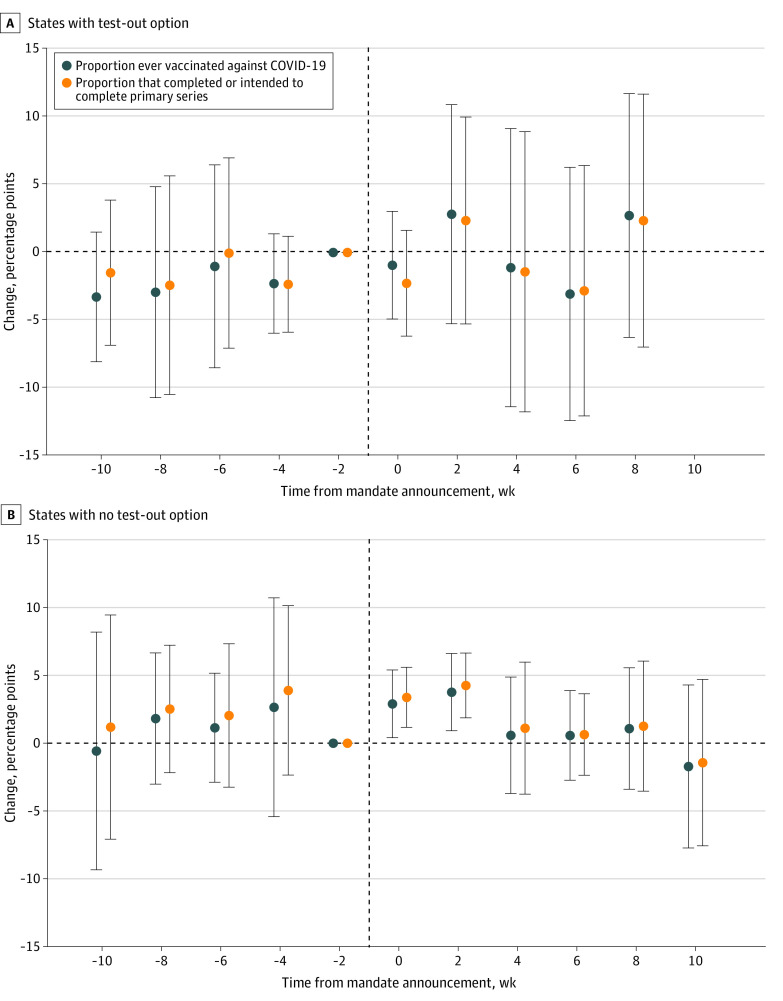
Event Study of the Association Between State COVID-19 Vaccine Mandates and Uptake Among Health Care Workers by Stringency of Mandates The plotted points are regression coefficients on a series of indicators for survey weeks before and after the announcement of state vaccine mandates, derived from a staggered difference-in-differences analysis described in the Methods section. The vertical dashed line represents the mandate announcement. All estimates are relative to the survey week immediately prior to the mandate announcement and were weighted via survey weights. A survey week spanned 2 calendar weeks. Error bars indicate 95% CIs. Models controlled for state fixed effects, survey week fixed effects, individual characteristics (sex, age, race, ethnicity, marital status, educational level, and income level), and the lagged intensity of the COVID-19 pandemic of each state. Standard errors were clustered at the state level. The postmandate period of states with a test-out option did not extend to event time 10. Additional details are given in eAppendix 3 in Supplement 1.

In the analyses stratified by mandate stringency, we found no statistically significant associations between mandates and vaccine uptake in states with a test-out option (Figure 2A). Regarding states with no test-out option, results revealed a 2.90-pp (95% CI, 0.32-5.49 pp; *P* = .03) increase in the proportion of HCWs ever vaccinated against COVID-19 in week 0 and week 1 and a 3.77-pp (95% CI, 0.82-6.71 pp; *P* = .01) increase in week 2 and week 3 after mandate announcement (Figure 2B). Relative to a baseline proportion of 87.35%, these represented increases of 3.32% and 4.31%, respectively. Associations were also observed for the proportion of HCWs who completed or intended to complete the primary series, with an increase of 3.38 pp (95% CI, 1.10-5.67 pp; *P* = .005), or 3.97% relative to the baseline proportion (85.09%), in week 0 and week 1 and an increase of 4.26 pp (95% CI, 1.79-6.73 pp; *P* = .001), or 5.01% relative to the baseline, in week 2 and week 3 after mandate announcement. Results by Callaway and Sant’Anna^23^ and TWFE estimators were generally consistent (eFigures 5-8 and eTables 4 and 5 in Supplement 1). In addition, as the scope of mandates may have varied across states, we also conducted a robustness check by excluding states with mandates only for long-term care facilities, hospitals, and/or state health care facilities. Findings were similar for the remaining states with broad mandate scope (eFigures 9 and 10 and eTable 6 in Supplement 1) compared with the main results. Further, in the stratified analysis using the new sample, positive associations were again only detected in mandate states with no test-out option (eFigures 11-14 and eTables 7 and 8 in Supplement 1).

**Figure 3.**
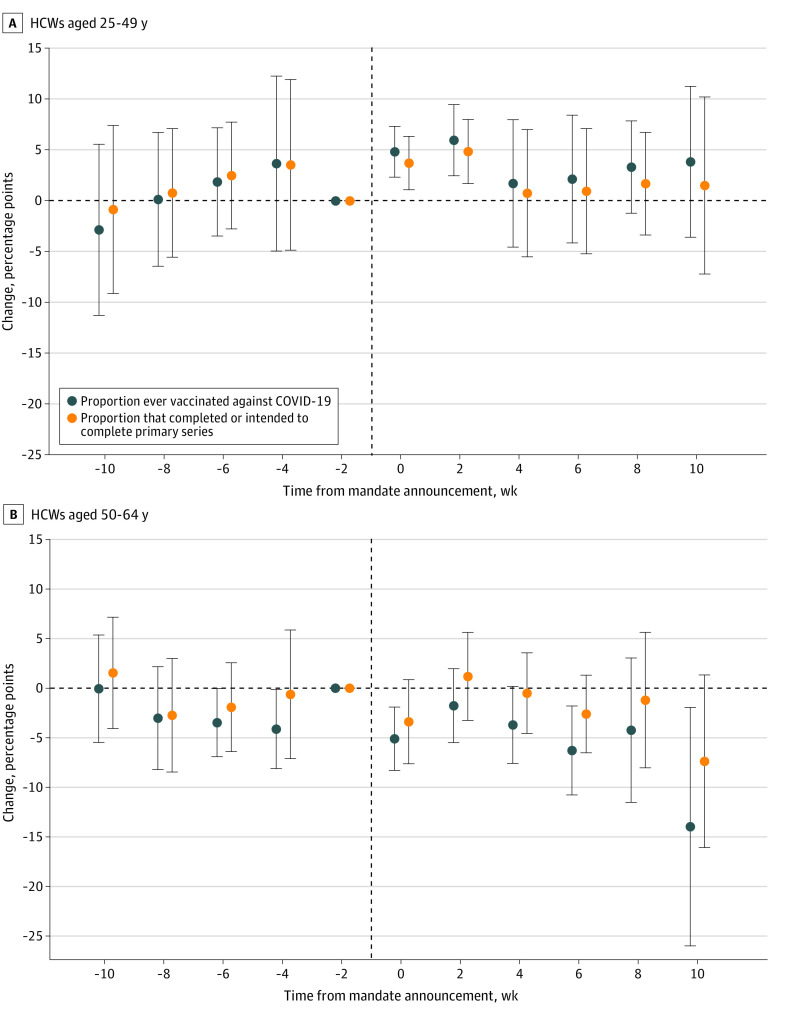
Event Study of the Association Between State COVID-19 Vaccine Mandates and Uptake Among Health Care Workers (HCWs) by Age The plotted points are regression coefficients on a series of indicators for survey weeks before and after the announcement of state vaccine mandates, derived from a staggered difference-in-differences analysis described in the Methods section. The vertical dashed line represents the mandate announcement. All estimates are relative to the survey week immediately prior to the mandate announcement and were weighted via survey weights. A survey week spanned 2 calendar weeks. Error bars indicate 95% CIs. Models controlled for state fixed effects, survey week fixed effects, individual characteristics (sex, age, race, ethnicity, marital status, educational level, and income levels), and the lagged intensity of the COVID-19 pandemic of each state. Standard errors were clustered at the state level. Additional details are given in eAppendix 3 in Supplement 1.

In the analyses stratified by HCW age, the vaccinated proportion of younger HCWs (aged 25-49 years) in mandate states increased substantially after mandate announcement compared with HCWs in nonmandate states (Figure 3A). Specifically, the mandates were associated with a 4.84-pp (95% CI, 2.26-7.41 pp; *P* < .001) increase in the proportion ever vaccinated against COVID-19 in week 0 and week 1 and a 5.97-pp (95% CI, 2.37-9.57 pp; *P* = .002) increase in week 2 and week 3 after mandate announcement, representing increases of 5.74% and 7.09%, respectively, relative to a baseline proportion of 84.26%. A smaller increase was observed for the proportion of HCWs that completed or intended to complete the primary series: 3.72 pp (95% CI, 1.02-6.43 pp; *P* = .01) in week 0 and week 1 and 4.86 pp (95% CI, 1.61-8.10 pp; *P* = .004) in week 2 and week 3 after mandate announcement. Relative to a baseline proportion of 83.98%, these correspond to increases of 4.43% and 5.78%, respectively. The estimates using the sample of older HCWs (aged 50-64 years) were either not significant or inconclusive due to violations of the parallel trends assumption (Figure 3B). Estimates using the Callaway and Sant’Anna^23^ and TWFE estimators revealed similar findings (eFigures 15-18 and eTables 9 and 10 in Supplement 1).

## Discussion

While a populationwide vaccine mandate could pose substantial ethical and political challenges, mandating COVID-19 vaccination for HCWs may be justified given its precedent in previous pandemics and the nature of the health care professions.^24,25^ In mid-2021, vaccine mandates were initiated after incentive-based strategies had been implemented; of the 16 studied states with mandates, 11 had implemented statewide COVID-19 vaccine lotteries, while the rest (Connecticut, New Jersey, Pennsylvania, Rhode Island, and Washington, DC) had other forms of vaccination incentive programs. After the US Supreme Court legally upheld the CMS COVID-19 vaccine mandate for HCWs in early 2022,^26^ nonmandate states started to follow suit. Some states even mandated booster shots for HCWs.^27^ Although these mandates have now been lifted following the decline of the COVID-19 pandemic, they serve as valuable examples of the potential effectiveness of immunization efforts for future pandemics.

Adding to the limited evidence on the impact of COVID-19 vaccine mandates in the US, this repeated cross-sectional analysis found that state mandates for HCWs introduced in mid-2021 were associated with a 2.90- to 5.97-pp increase in proportions of vaccinated HCWs from baseline proportions ranging from 83.98% to 87.98%. The increase was manifested in the first 4 weeks following mandate announcement and dissipated in later periods. Moreover, statistically significant associations were detected only in states with no test-out option and among younger HCWs (aged 25 to 49 years).

Our estimates for the postmandate period revealed the evolving changes in proportions of vaccinated HCWs in mandate states compared with those in nonmandate states relative to the baseline period. As HCWs in nonmandate states may have also experienced employer vaccine mandates prompted by callings from health care associations and the federal government before the CMS nationwide mandate released on November 4, 2021,^7^ our estimates may be more accurately interpreted as the potential incremental effects of state vaccine mandates compared with possible mandates from other sources. This may also explain why the increases in vaccination uptake started to dissipate in week 4 and week 5 in the postmandate period; this timing coincided with early September 2021, when the CMS restated its intention to mandate COVID-19 vaccination for HCWs in all Medicare- and Medicaid-funded health care facilities,^6^ which may have prompted more health care facilities in nonmandate states to implement mandates. Media evidence also showed that a number of health systems in nonmandate states required their staff to get vaccinated with deadlines from late September to early December 2021.^28^

Our results showed rapid effects of COVID-19 vaccine mandates, as also evidenced by prior research on mandates for nursing home workers in the US^13^ and vaccine passport mandates in Canada and Europe^29,30,31^; these studies found that vaccine uptake among specific populations rose immediately in the first few weeks following mandate announcement. Our results also suggest that the mandates had potential to promote both vaccine initiation and primary series completion among a population already broadly vaccinated (83.98%). Additionally, the mandates were associated with greater vaccine uptake among younger HCWs than their older counterparts. Consistent findings were reported from research on employer vaccine mandates for HCWs^32^ and vaccine passport mandates for the general population.^16,31,33^

Our study also suggested that a level of stringency may be necessary to ensure the effectiveness of mandates, as positive associations were detected only in mandate states with no test-out option in the stratified analysis. Similarly, Syme et al^12^ revealed that the vaccinated proportion of nursing home staff in Mississippi remained numerically similar to that in comparison states under a vaccinate-or-test policy. McGarry et al^13^ studied nursing home worker mandates in the US and found greater increases in vaccine uptake in states with no test-out option. Another study on the COVID-19 vaccine mandate for municipal employees in New York City also showed that the mandate effects became evident only after the previous test-out option was removed.^14^

### Limitations

This study has several limitations. First, as sampled HCWs were different in each survey wave, the data may contain larger sampling errors than if panel data were available. To address this concern, we controlled for individual-level covariates and survey-week fixed effects in the model and incorporated individual-level survey weights in all analyses. Second, as the HCWs in control states may have also experienced employer vaccine mandates or other mandates in the intervention period, our estimates could be interpreted as the potential incremental effects of state COVID-19 vaccine mandates beyond existing mandates from other organizations. Because of this, our results may serve as a conservative estimate for the association between state COVID-19 vaccine mandates and HCW vaccine uptake. Third, as the terminology for specific health care facilities was not uniform across official documents, the scope of vaccine mandates could have varied by state to different extents. Given this possible heterogeneity, we conducted a robustness check focusing on mandate states only with broad mandate scope. The results suggested findings similar to those using the full sample. Fourth, our vaccine uptake measures were based on self-reported vaccination status from survey respondents and were thus subject to self-report bias.

## Conclusions

This repeated cross-sectional study found that state COVID-19 vaccine mandates for HCWs introduced in mid-2021 were associated with an increase of 2.90 to 5.97 pp in the proportion of HCWs ever vaccinated against COVID-19 and an increase of 3.38 to 4.86 pp in the proportion who completed or intended to complete their primary vaccination series in mandate states compared with nonmandate states relative to baseline vaccinated proportions at or above 83.98% in each scenario. Increases were manifested in the first 4 weeks following mandate announcement. In the stratified analyses, positive associations were only detected in states with no test-out option and among younger HCWs. The study demonstrated the potential for vaccine mandates to further promote vaccine uptake among a broadly vaccinated population, especially when a level of mandate stringency is maintained.

## Notes

Supplement 1. **eAppendix 1.** Sample States **eTable 1.** Summary of the HCW Mandates in the Study **eAppendix 2.** Methods **eAppendix 3.** Analytical Results **eFigure 1.** Event Study of Association Between State COVID-19 Vaccine Mandates and Proportion of HCWs Ever Vaccinated Against COVID-19, Full Sample Unadjusted for Covariates **eFigure 2**. Event Study of Association Between State COVID-19 Vaccine Mandates and Proportion of HCWs Ever Vaccinated Against COVID-19, Full Sample Adjusted for Covariates **eTable 2.** Estimates of Association Between State COVID-19 Vaccine Mandates and Proportion of HCWs Ever Vaccinated Against COVID-19, Full Sample **eFigure 3.** Event Study of Association Between State COVID-19 Vaccine Mandates and Proportion of HCWs Who Completed or Intended to Complete the Primary Series, Full Sample Unadjusted for Covariates **eFigure 4**. Event Study of Association Between State COVID-19 Vaccine Mandates and Proportion of HCWs Who Completed or Intended to Complete the Primary Series, Full Sample Adjusted for Covariates **eTable 3.** Estimates of the Association Between State COVID-19 Vaccine Mandates and Proportion of HCWs Who Completed or Intended to Complete the Primary Series, Full Sample **eFigure 5.** Event Study of Association Between State COVID-19 Vaccine Mandates and Proportion of HCWs Ever Vaccinated Against COVID-19, States With a Test-Out Option **eFigure 6.** Event Study of Association Between State COVID-19 Vaccine Mandates and Proportion of HCWs Who Completed or Intended to Complete the Primary Series, States With a Test-Out Option **eTable 4.** Estimates of Associations Between State COVID-19 Vaccine Mandates and Vaccine Uptake Among HCWs, States With a Test-Out Option **eFigure 7.** Event Study of Association Between State COVID-19 Vaccine Mandates and Proportion of HCWs Ever Vaccinated Against COVID-19, States With No Test-Out Option **eFigure 8.** Event Study of Association Between State COVID-19 Vaccine Mandates and Proportion of HCWs Who Completed or Intended to Complete the Primary Series, States With No Test-Out Option **eTable 5.** Estimates of Associations Between State COVID-19 Vaccine Mandates and Vaccine Uptake Among HCWs, States With No Test-Out Option **eFigure 9.** Event Study of Association Between State COVID-19 Vaccine Mandates and Proportion of HCWs Ever Vaccinated Against COVID-19, States With Broad Mandate Scope **eFigure 10.** Event Study of Association Between State COVID-19 Vaccine Mandates and Proportion of HCWs Who Completed or Intended to Complete the Primary Series, States With Broad Mandate Scope **eTable 6.** Estimates of Associations Between State COVID-19 Vaccine Mandates and Vaccine Uptake Among HCWs, States With Broad Mandate Scope **eFigure 11.** Event Study of Association Between State COVID-19 Vaccine Mandates and Proportion of HCWs Ever Vaccinated Against COVID-19, States With Broad Mandate Scope and a Test-Out Option **eFigure 12.** Event Study of Association Between State COVID-19 Vaccine Mandates and Proportion of HCWs Who Completed or Intended to Complete the Primary Series, States With Broad Mandate Scope and a Test-Out Option **eTable 7.** Estimates of Associations Between State COVID-19 Vaccine Mandates and Vaccine Uptake Among HCWs, States With Broad Mandate Scope and a Test-Out Option **eFigure 13.** Event Study of Association Between State COVID-19 Vaccine Mandates and Proportion of HCWs Ever Vaccinated Against COVID-19, States With Broad Mandate Scope and No Test-Out Option **eFigure 14.** Event Study of Association Between State COVID-19 Vaccine Mandates and Proportion of HCWs Who Completed or Intended to Complete the Primary Series, States With Broad Mandate Scope and No Test-Out Option **eTable 8.** Estimates of Associations Between State COVID-19 Vaccine Mandates and Vaccine Uptake Among HCWs, States With Broad Mandate Scope and No Test-Out Option **eFigure 15.** Event Study of Association Between State COVID-19 Vaccine Mandates and Proportion of HCWs Ever Vaccinated Against COVID-19, HCWs Aged 25-49 Years **eFigure 16.** Event Study of Association Between State COVID-19 Vaccine Mandates and Proportion of HCWs Who Completed or Intended to Complete the Primary Series, HCWs Aged 25-49 Years **eTable 9.** Estimates of Associations Between State COVID-19 Vaccine Mandates and Vaccine Uptake Among HCWs Aged 25-49 Years **eFigure 17.** Event Study of Association Between State COVID-19 Vaccine Mandates and Proportion of HCWs Ever Vaccinated Against COVID-19, HCWs Aged 50-64 Years **eFigure 18.** Event Study of Association Between State COVID-19 Vaccine Mandates and Proportion of HCWs Who Completed or Intended to Complete the Primary Series, HCWs Aged 50-64 Years **eTable 10.** Estimates of Associations Between State COVID-19 Vaccine Mandates and Vaccine Uptake Among HCWs Aged 50-64 Years
eReferences

Supplement 2.
Data Sharing Statement
