# Supplementary material for: State COVID-19 Vaccine Mandates and Uptake Among Health Care Workers in the US
Source: JAMA Netw Open. 2024 Aug 14;7(8):e2426847. doi: 10.1001/jamanetworkopen.2024.26847 (PMC11325213; doi:10.1001/jamanetworkopen.2024.26847)
Supplement: Supplement 1. — eAppendix 1. Sample States eTable 1. Summary of the HCW Mandates in the Study eAppendix 2. Methods eAppendix 3. Analytical Results eFigure 1. Event Study of Association Between State COVID-19 Vaccine Mandates and Proportion of HCWs Ever Vaccinated Against COVID-19, Full Sample Unadjusted for Covariates eFigure 2. Event Study of Association Between State COVID-19 Vaccine Mandates and Proportion of HCWs Ever Vaccinated Against COVID-19, Full Sample Adjusted for Covariates eTable 2. Estimates of Association Between State COVID-19 Vaccine Mandates and Proportion of HCWs Ever Vaccinated Against COVID-19, Full Sample eFigure 3. Event Study of Association Between State COVID-19 Vaccine Mandates and Proportion of HCWs Who Completed or Intended to Complete the Primary Series, Full Sample Unadjusted for Covariates eFigure 4. Event Study of Association Between State COVID-19 Vaccine Mandates and Proportion of HCWs Who Completed or Intended to Complete the Primary Series, Full Sample Adjusted for Covariates eTable 3. Estimates of the Association Between State COVID-19 Vaccine Mandates and Proportion of HCWs Who Completed or Intended to Complete the Primary Series, Full Sample eFigure 5. Event Study of Association Between State COVID-19 Vaccine Mandates and Proportion of HCWs Ever Vaccinated Against COVID-19, States With a Test-Out Option eFigure 6. Event Study of Association Between State COVID-19 Vaccine Mandates and Proportion of HCWs Who Completed or Intended to Complete the Primary Series, States With a Test-Out Option eTable 4. Estimates of Associations Between State COVID-19 Vaccine Mandates and Vaccine Uptake Among HCWs, States With a Test-Out Option eFigure 7. Event Study of Association Between State COVID-19 Vaccine Mandates and Proportion of HCWs Ever Vaccinated Against COVID-19, States With No Test-Out Option eFigure 8. Event Study of Association Between State COVID-19 Vaccine Mandates and Proportion of HCWs Who Completed or Intended to Complete the Primary Series, S [file jamanetwopen-e2426847-s001.pdf]

Supplementary Online Content

Wang Y, Stoecker C, Callison K, Hernandez JH. State COVID-19 vaccine mandates and uptake among health care workers in the US. *JAMA Netw Open*. 2024;7(8):e2426847. doi:10.1001/jamanetworkopen.2024.26847

eAppendix 1. Sample States

eTable 1. Summary of the HCW Mandates in the Study

eAppendix 2. Methods

eAppendix 3. Analytical Results

eFigure 1. Event Study of Association Between State COVID-19 Vaccine Mandates and Proportion of HCWs Ever Vaccinated Against COVID-19, Full Sample Unadjusted for Covariates

eFigure 2. Event Study of Association Between State COVID-19 Vaccine Mandates and Proportion of HCWs Ever Vaccinated Against COVID-19, Full Sample Adjusted for Covariates

eTable 2. Estimates of Association Between State COVID-19 Vaccine Mandates and Proportion of HCWs Ever Vaccinated Against COVID-19, Full Sample

eFigure 3. Event Study of Association Between State COVID-19 Vaccine Mandates and Proportion of HCWs Who Completed or Intended to Complete the Primary Series, Full Sample Unadjusted for Covariates

eFigure 4. Event Study of Association Between State COVID-19 Vaccine Mandates and Proportion of HCWs Who Completed or Intended to Complete the Primary Series, Full Sample Adjusted for Covariates

eTable 3. Estimates of the Association Between State COVID-19 Vaccine Mandates and Proportion of HCWs Who Completed or Intended to Complete the Primary Series, Full Sample

eFigure 5. Event Study of Association Between State COVID-19 Vaccine Mandates and Proportion of HCWs Ever Vaccinated Against COVID-19, States With a Test-Out Option

eFigure 6. Event Study of Association Between State COVID-19 Vaccine Mandates and Proportion of HCWs Who Completed or Intended to Complete the Primary Series, States With a Test-Out Option

© 2024 Wang Y et al. *JAMA Network Open.*

eTable 4. Estimates of Associations Between State COVID-19 Vaccine Mandates and Vaccine Uptake Among HCWs, States With a Test-Out Option

eFigure 7. Event Study of Association Between State COVID-19 Vaccine Mandates and Proportion of HCWs Ever Vaccinated Against COVID-19, States With No Test-Out Option

eFigure 8. Event Study of Association Between State COVID-19 Vaccine Mandates and Proportion of HCWs Who Completed or Intended to Complete the Primary Series, States With No Test-Out Option

eTable 5. Estimates of Associations Between State COVID-19 Vaccine Mandates and Vaccine Uptake Among HCWs, States With No Test-Out Option

eFigure 9. Event Study of Association Between State COVID-19 Vaccine Mandates and Proportion of HCWs Ever Vaccinated Against COVID-19, States With Broad Mandate Scope

eFigure 10. Event Study of Association Between State COVID-19 Vaccine Mandates and Proportion of HCWs Who Completed or Intended to Complete the Primary Series, States With Broad Mandate Scope

eTable 6. Estimates of Associations Between State COVID-19 Vaccine Mandates and Vaccine Uptake Among HCWs, States With Broad Mandate Scope

eFigure 11. Event Study of Association Between State COVID-19 Vaccine Mandates and Proportion of HCWs Ever Vaccinated Against COVID-19, States With Broad Mandate Scope and a Test-Out Option

eFigure 12. Event Study of Association Between State COVID-19 Vaccine Mandates and Proportion of HCWs Who Completed or Intended to Complete the Primary Series, States With Broad Mandate Scope and a Test-Out Option

eTable 7. Estimates of Associations Between State COVID-19 Vaccine Mandates and Vaccine Uptake Among HCWs, States With Broad Mandate Scope and a Test-Out Option

eFigure 13. Event Study of Association Between State COVID-19 Vaccine Mandates and Proportion of HCWs Ever Vaccinated Against COVID-19, States With Broad Mandate Scope and No Test-Out Option

eFigure 14. Event Study of Association Between State COVID-19 Vaccine Mandates and Proportion of HCWs Who Completed or Intended to Complete the Primary Series, States With Broad Mandate Scope and No Test-Out Option

eTable 8. Estimates of Associations Between State COVID-19 Vaccine Mandates and Vaccine Uptake Among HCWs, States With Broad Mandate Scope and No Test-Out Option

© 2024 Wang Y et al. *JAMA Network Open.*

eFigure 15. Event Study of Association Between State COVID-19 Vaccine Mandates and Proportion of HCWs Ever Vaccinated Against COVID-19, HCWs Aged 25-49 Years

eFigure 16. Event Study of Association Between State COVID-19 Vaccine Mandates and Proportion of HCWs Who Completed or Intended to Complete the Primary Series, HCWs Aged 25-49 Years

eTable 9. Estimates of Associations Between State COVID-19 Vaccine Mandates and Vaccine Uptake Among HCWs Aged 25-49 Years

eFigure 17. Event Study of Association Between State COVID-19 Vaccine Mandates and Proportion of HCWs Ever Vaccinated Against COVID-19, HCWs Aged 50-64 Years

eFigure 18. Event Study of Association Between State COVID-19 Vaccine Mandates and Proportion of HCWs Who Completed or Intended to Complete the Primary Series, HCWs Aged 50-64 Years

eTable 10. Estimates of Associations Between State COVID-19 Vaccine Mandates and Vaccine Uptake Among HCWs Aged 50-64 Years

eReferences

This supplementary material has been provided by the authors to give readers additional information about their work.

eAppendix 1. Sample States

We collected COVID-19 vaccine mandate policies for HCWs from the Thomson Reuters Practical Law database for Vaccine Mandates and Prohibitions[1] and further checked mandate requirements through state news releases, memoranda, and related websites. Six states were excluded from the study because (1) Hawaii,[2] Minnesota,[3] and Nevada[4] implemented state employee mandates but not HCW mandates, which may have indirectly affected HCWs; (2) Kentucky only “strongly encouraged” workers in state-run healthcare facilities to get vaccinated;[5] (3) Idaho rescinded the vaccine mandate shortly after its enactment;[6] and (4) Mississippi announced a vaccine mandate for nursing home workers on June 14, 2021, more than a month ahead of other states, and was only in effect until September 30, 2021.[7] In addition, government documents could use varied terminologies to describe similar mandate scope. We extracted the core terminology from the original documents to showcase the main scope of mandates in eTable 1. A state was further coded as having a test-out option when the government document or news release explicitly mentioned the availability of a test-out option.

eTable 1. Summary of the HCW Mandates in the Study

State	Main scope	Test-out option	Date of announcement
CA[8,9]	Healthcare facilities, high-risk congregate settings, in-home direct care settings	No	July 26, 2021[a]
CO[10]	Healthcare facilities	No	August 17, 2021
CT[11]	Long-term care facilities, state hospitals	No	August 6, 2021
DE[12]	Acute and outpatient providers, hospitals, home health agencies, long-term care facilities	Yes	August 12, 2021
DC[13]	Healthcare facilities	No	August 16, 2021
IL[14]	Healthcare facilities	Yes	August 26, 2021
ME[15]	Healthcare facilities[b]	No	August 12, 2021
MD[16]	Hospitals, nursing homes	Yes	August 18, 2021
MA[17]	Skilled nursing facilities, soldiers' homes, assisted living facilities	No	August 4, 2021
NJ[18]	Healthcare facilities, high-risk congregate settings	Yes	August 2, 2021
NM[19]	Hospitals, congregate care facilities	No	August 17, 2021
NY[20,21]	Healthcare facilities	No	July 28, 2021
OR[22,23]	Healthcare facilities	No	August 4, 2021
PA[24]	State healthcare facilities, high-risk congregate care facilities	Yes	August 10, 2021
RI[25,26]	RIDOH-licensed healthcare facilities, RIDOH-licensed healthcare providers	No	August 18, 2021
WA[27]	Public and private healthcare providers	No	August 9, 2021

Note.

Mississippi was excluded from the study. The state implemented a nursing home worker COVID-19 vaccine mandate with a test-out option.

a. The governor announced the mandate on July 26, 2021, while the official document was released on August 5, 2021.

© 2024 Wang Y et al. *JAMA Network Open*.

b. Dentists and Emergency Medical Services (EMS) personnel were later removed from the targeted group as they had developed their own COVID-19 vaccine mandates.

© 2024 Wang Y et al. *JAMA Network Open*.

eAppendix 2. Methods

To deal with dynamic mandate adoptions and heterogeneous treatment effects across the mandate states, we established our main specification based on a staggered difference-in-differences method proposed by Sun and Abraham,[28] which was specifically built on the event study design. The method groups mandate states into cohorts according to the survey week during which a mandate was announced, and the final treatment effect for each relative time period is the weighted average of all cohort-specific average treatment effects on the treated (CATTs) of that relative period. The CATT is estimated through the following specification:

$$Y_{ist} = \alpha + \sum_{e=34}^{36} \sum_{l=-5,l\neq-1}^{5} \beta_{el} \left(\mathbf{1}\{E_s = e\} \cdot \mathbf{1}\{t - E_s = l\}\right) + \gamma X_{ist} + \delta Z_{s,t-1} + \mu_s + \tau_t + \varepsilon_{ist} \qquad (1)$$

where Y_{ist} represents the outcome of individual i in state s at survey week t; E_s denotes the survey week when state s announced the HCW vaccine mandate and equals $e \in$ {34, 35, 36} in our study; l indicates the relative survey weeks leading up to and following the survey week of mandate announcement; β_{el} denotes the CATT at the relative survey week l for the state cohort whose E_s equals e; X_{ist} is a collection of individual socio-demographic covariates mentioned in the main text; $Z_{s,t-1}$ denotes the lagged intensity of the COVID-19 pandemic of each state, measured by COVID-19 mortality rates of each state in the bi-week before each survey week (COVID-19 death counts within that bi-week divided by the state's population in 2021); μ_s and τ_t are state and survey week fixed effect terms, respectively. The standard errors were clustered at the state level to account for the within-state serial correlation in vaccine uptake over time.

The final treatment effect for a relative period is estimated by the formula:

$$\nu_g = \frac{1}{|g|} \sum_{l\in g} \sum_{e} \beta_{el} Pr\{E_i = e \mid E_i \in [34, 36]\} \qquad (2)$$

where g denotes all possible values of relative survey week l; ν_g represents the final treatment effect for the relative survey week $l \in g$; $|g|$ indicates the size of g; $Pr\{E_i = e \mid E_i \in [34, 36]\}$ is the weight estimated by the sample share of each cohort in the relative survey week l.

© 2024 Wang Y et al. *JAMA Network Open*.

eAppendix 3. Analytical Results

This section exhibits event study estimates of the association between state COVID-19 vaccine mandates for HCWs and vaccine uptake among this population, derived from 3 difference-in-differences estimators (i.e., Sun & Abraham (S&A) estimator,[28] Callaway & Sant'Anna (C&S) estimator,[29] and 2-way fixed effects (TWFE) estimator), using the biweekly individual-level data from the Household Pulse Survey (HPS). All estimates are relative to the survey week immediately prior to the announcement of state vaccine mandates and were weighted via survey weights.

© 2024 Wang Y et al. *JAMA Network Open*.

3.1 Main Analysis

3.1.1 Results for Outcome 1

eFigure 1. Event Study of Association Between State COVID-19 Vaccine Mandates and Proportion of HCWs Ever Vaccinated against COVID-19, Full Sample Unadjusted for Covariates

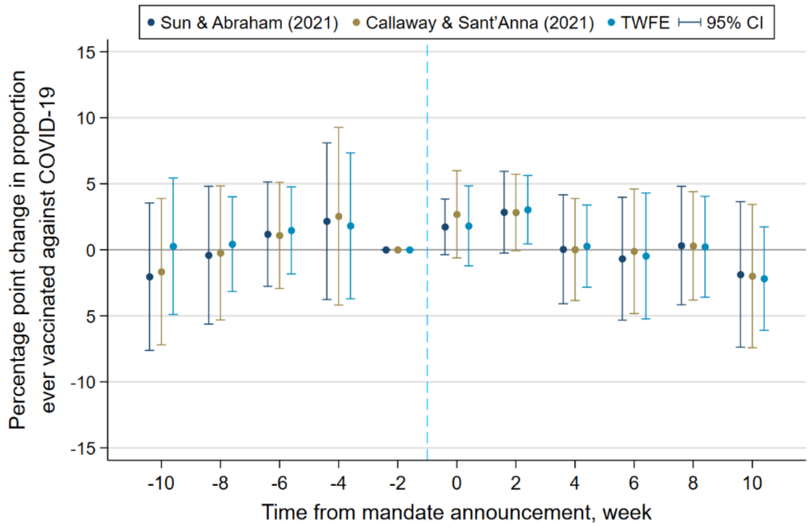

Note. Estimates with 95% CIs were derived from 3 difference-in-differences methods mentioned above using the biweekly HPS data with survey weights applied. Estimates here were not adjusted for covariates.

© 2024 Wang Y et al. *JAMA Network Open*.

eFigure 2. Event Study of Association Between State COVID-19 Vaccine Mandates and Proportion of HCWs Ever Vaccinated against COVID-19, Full Sample Adjusted for Covariates

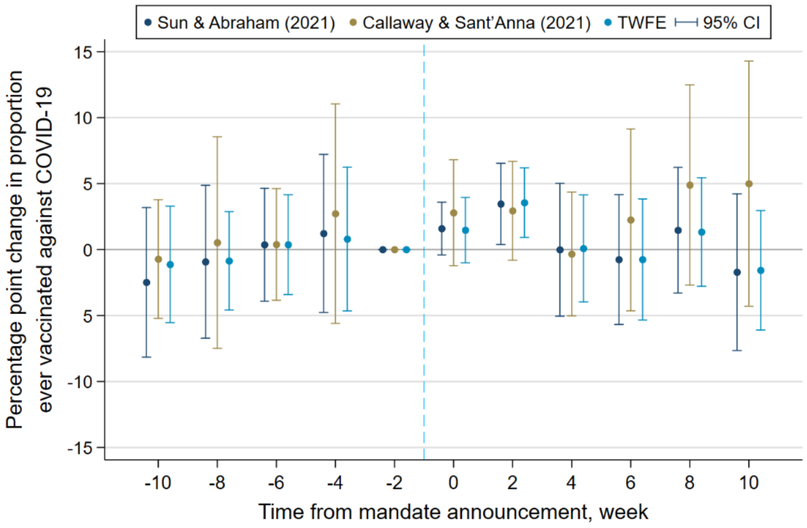

Note. Estimates with 95% CIs were derived from 3 difference-in-differences methods mentioned above using the biweekly HPS data with survey weights applied. Estimates here were adjusted for covariates described in the main text.

© 2024 Wang Y et al. *JAMA Network Open*.

eTable 2. Estimates of the Association Between State COVID-19 Vaccine Mandates and Proportion of HCWs Ever Vaccinated against COVID-19, Full Sample

	Unadjusted for covariates			Adjusted for covariates		
	**S&A**	**C&S**	**TWFE**	**S&A**	**C&S**	**TWFE**
Event time 0	1.73	2.69	1.81	1.59	2.79	1.47
	[-0.43, 3.90]	[-0.62, 5.99]	[-1.30, 4.92]	[-0.46, 3.65]	[-1.22, 6.80]	[-1.07, 4.02]
	(0.11)	(0.11)	(0.25)	(0.13)	(0.17)	(0.25)
Event time 2	2.85*	2.83*	3.04**	3.46**	2.94	3.56**
	[-0.33, 6.03]	[-0.07, 5.72]	[0.37, 5.70]	[0.29, 6.63]	[-0.81, 6.69]	[0.85, 6.26]
	(0.08)	(0.06)	(0.03)	(0.03)	(0.12)	(0.01)
Baseline outcome level (%)	87.98					
Policy effect at time 0 (%)	1.97	3.05	2.06	1.81	3.17	1.67
Policy effect at time 2 (%)	3.24	3.21	3.45	3.93	3.34	4.04
N	31 142					

Note. Estimates were based on biweekly data. The 95% confidence interval (CI) in square brackets; the *P* value in parentheses. The “baseline outcome level” is the weighted mean of the outcome variable in the period immediately prior to mandate announcement. The “policy effect (%)” equals the estimate for the corresponding period divided by the baseline outcome level. For brevity purposes, the table only presents estimates for event times 0 and 2. Estimates for other periods are available upon request.

** $p<0.05$, * $p<0.1$

© 2024 Wang Y et al. *JAMA Network Open.*

3.1.2 Results for Outcome 2

eFigure 3. Event Study of Association Between State COVID-19 Vaccine Mandates and Proportion of HCWs Who Completed or Intended to Complete the Primary Series, Full Sample Unadjusted for Covariates

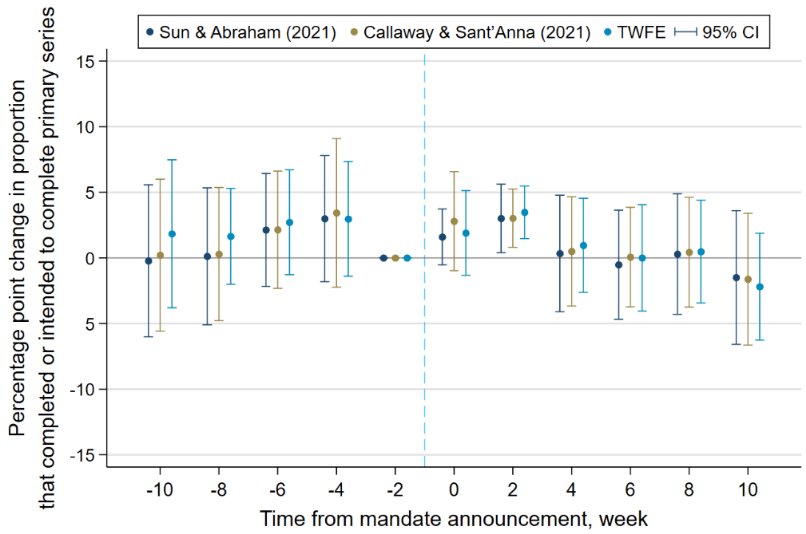

Note. Estimates with 95% CIs were derived from 3 difference-in-differences methods mentioned above using the biweekly HPS data with survey weights applied. Estimates here were not adjusted for covariates.

© 2024 Wang Y et al. *JAMA Network Open.*

eFigure 4. Event Study of Association Between State COVID-19 Vaccine Mandates and Proportion of HCWs Who Completed or Intended to Complete the Primary Series, Full Sample Adjusted for Covariates

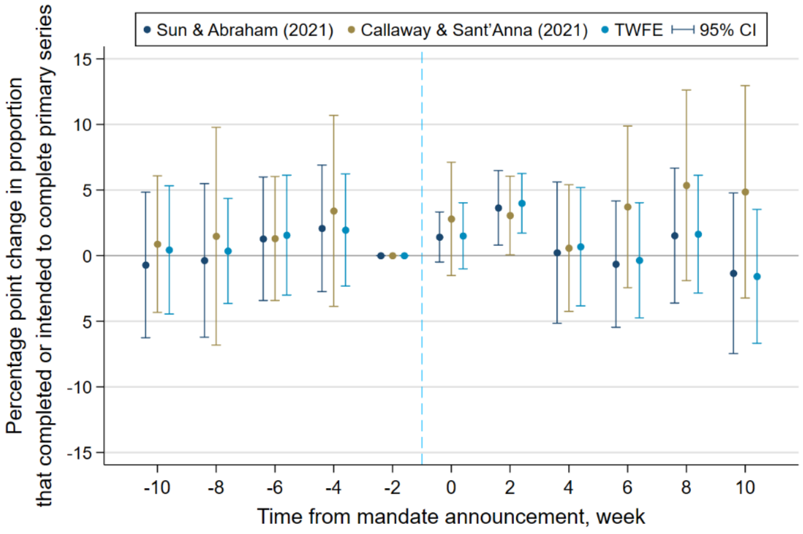

Note. Estimates with 95% CIs were derived from 3 difference-in-differences methods mentioned above using the biweekly HPS data with survey weights applied. Estimates here were adjusted for covariates described in the main text.

© 2024 Wang Y et al. *JAMA Network Open*.

eTable 3. Estimates of the Association Between State COVID-19 Vaccine Mandates and Proportion of HCWs Who Completed or Intended to Complete the Primary Series, Full Sample

	Unadjusted for covariates			Adjusted for covariates		
	**S&A**	**C&S**	**TWFE**	**S&A**	**C&S**	**TWFE**
Event time 0	1.59	2.80	1.90	1.42	2.80	1.51
	[-0.60, 3.78]	[-0.98, 6.57]	[-1.42, 5.22]	[-0.54, 3.38]	[-1.51, 7.12]	[-1.08, 4.10]
	(0.15)	(0.15)	(0.26)	(0.15)	(0.20)	(0.25)
Event time 2	3.01**	3.02***	3.48***	3.64**	3.06**	3.99***
	[0.33, 5.69]	[0.80, 5.24]	[1.42, 5.53]	[0.72, 6.57]	[0.07, 6.06]	[1.66, 6.33]
	(0.03)	(0.008)	(0.001)	(0.02)	(0.05)	(0.001)
Baseline outcome level (%)	86.12					
Policy effect at time 0 (%)	1.85	3.25	2.21	1.64	3.25	1.75
Policy effect at time 2 (%)	3.49	3.51	4.04	4.23	3.55	4.64
N	31 142					

Note. Estimates were based on biweekly data. The 95% confidence interval (CI) in square brackets; the *P* value in parentheses. The "baseline outcome level" is the weighted mean of the outcome variable in the period immediately prior to mandate announcement. The "policy effect (%)" equals the estimate for the corresponding period divided by the baseline outcome level. For brevity purposes, the table only presents estimates for event times 0 and 2. Estimates for other periods are available upon request.

*** $p<0.01$, ** $p<0.05$

© 2024 Wang Y et al. *JAMA Network Open*.

3.2 Stratified Analysis by Stringency of Mandates

3.2.1 Results for States with a Test-out Option

eFigure 5. Event Study of Association Between State COVID-19 Vaccine Mandates and Proportion of HCWs Ever Vaccinated against COVID-19, States with a Test-out Option

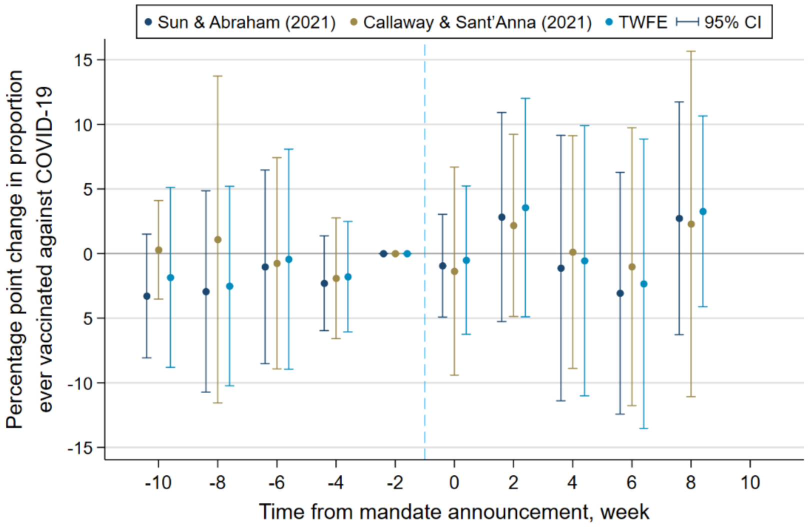

Note. Estimates with 95% CIs were derived from 3 difference-in-differences methods mentioned above using the biweekly HPS data with survey weights applied. Estimates here were adjusted for covariates. The post-mandate period of this subgroup did not extend to event time 10.

© 2024 Wang Y et al. *JAMA Network Open.*

eFigure 6. Event Study of Association Between State COVID-19 Vaccine Mandates and Proportion of HCWs Who Completed or Intended to Complete the Primary Series, States with a Test-out Option

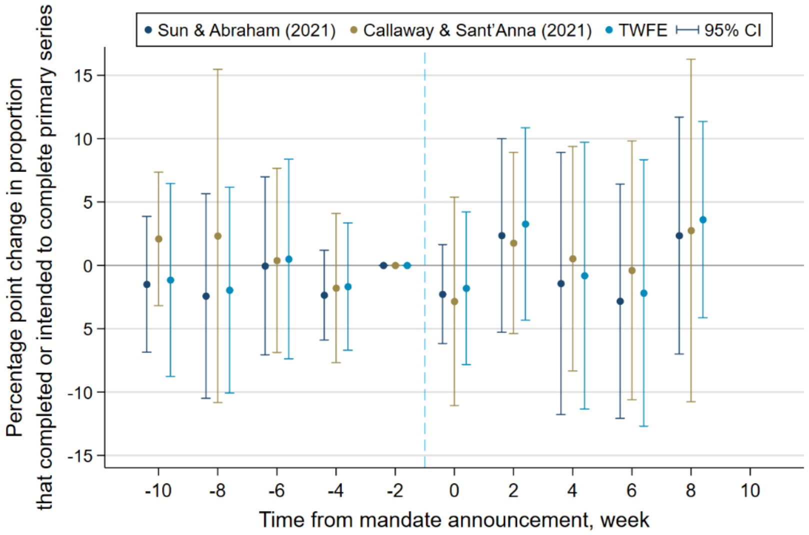

Note. Estimates with 95% CIs were derived from 3 difference-in-differences methods mentioned above using the biweekly HPS data with survey weights applied. Estimates here were adjusted for covariates. The post-mandate period of this subgroup did not extend to event time 10.

© 2024 Wang Y et al. *JAMA Network Open*.

eTable 4. Estimates of Associations Between State COVID-19 Vaccine Mandates and Vaccine Uptake Among HCWs, States with a Test-out Option

	Outcome 1			Outcome 2		
	S&A	C&S	TWFE	S&A	C&S	TWFE
Event time 0	-0.94	-1.36	-0.51	-2.27	-2.84	-1.81
	[-5.06, 3.18]	[-9.42, 6.69]	[-6.46, 5.44]	[-6.33, 1.78]	[-11.07, 5.39]	[-8.06, 4.45]
	(0.65)	(0.74)	(0.86)	(0.26)	(0.50)	(0.56)
Event time 2	2.82	2.18	3.56	2.36	1.77	3.27
	[-5.57, 11.22]	[-4.87, 9.22]	[-5.21, 12.33]	[-5.57, 10.29]	[-5.38, 8.92]	[-4.61, 11.15]
	(0.50)	(0.54)	(0.42)	(0.55)	(0.63)	(0.40)
Baseline outcome level (%)		89.95			89.31	
N			22 146			

Note. Estimates were based on biweekly data and were adjusted for covariates. The "outcome 1" corresponds to proportion of HCWs ever vaccinated against COVID-19; the "outcome 2" corresponds to proportion of HCWs who completed or intended to complete the primary series. The 95% confidence interval (CI) in square brackets; the *P* value in parentheses. The "baseline outcome level" is the weighted mean of the outcome variable in the period immediately before mandate announcement. For brevity purposes, the table only presents estimates for event times 0 and 2. Estimates for other periods are available upon request.

© 2024 Wang Y et al. *JAMA Network Open*.

3.2.2 Results for States with No Test-out Option

eFigure 7. Event Study of Association Between State COVID-19 Vaccine Mandates and Proportion of HCWs Ever Vaccinated against COVID-19, States with No Test-out Option

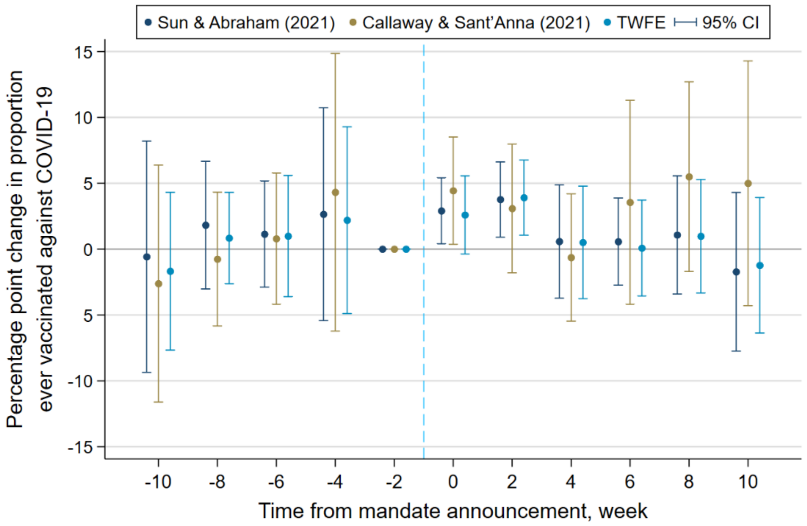

Note. Estimates with 95% CIs were derived from 3 difference-in-differences methods mentioned above using the biweekly HPS data with survey weights applied. Estimates here were adjusted for covariates described in the main text.

© 2024 Wang Y et al. *JAMA Network Open*.

eFigure 8. Event Study of Association Between State COVID-19 Vaccine Mandates and Proportion of HCWs Who Completed or Intended to Complete the Primary Series, States with No Test-out Option

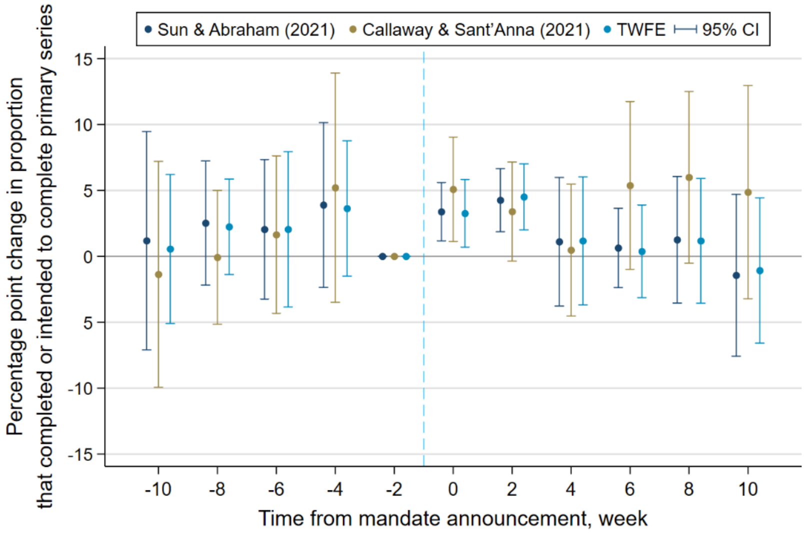

Note. Estimates with 95% CIs were derived from 3 difference-in-differences methods mentioned above using the biweekly HPS data with survey weights applied. Estimates here were adjusted for covariates described in the main text.

© 2024 Wang Y et al. *JAMA Network Open*.

eTable 5. Estimates of Associations Between State COVID-19 Vaccine Mandates and Vaccine Uptake Among HCWs, States with No Test-out Option

	Outcome 1			Outcome 2		
	**S&A**	**C&S**	**TWFE**	**S&A**	**C&S**	**TWFE**
Event time 0	2.90**	4.44**	2.60*	3.38***	5.08**	3.26**
	[0.32, 5.49]	[0.36, 8.51]	[-0.46, 5.66]	[1.10, 5.67]	[1.12, 9.04]	[0.61, 5.91]
	(0.03)	(0.03)	(0.09)	(0.005)	(0.01)	(0.02)
Event time 2	3.77**	3.08	3.91**	4.26***	3.40*	4.51***
	[0.82, 6.71]	[-1.80, 7.97]	[0.96, 6.85]	[1.79, 6.73]	[-0.36, 7.16]	[1.92, 7.09]
	(0.01)	(0.22)	(0.01)	(0.001)	(0.08)	(0.001)
Baseline outcome level (%)		87.35			85.09	
Policy effect at time 0 (%)	3.32	5.08	2.97	3.97	5.97	3.83
Policy effect at time 2 (%)	4.31	3.53	4.47	5.01	3.99	5.30
N			27 707			

Note. Estimates were based on biweekly data and were adjusted for covariates. The "outcome 1" corresponds to proportion of HCWs ever vaccinated against COVID-19; the "outcome 2" corresponds to proportion of HCWs who completed or intended to complete the primary series. The 95% confidence interval (CI) in square brackets; the *P* value in parentheses. The "baseline outcome level" is the weighted mean of the outcome variable in the period immediately before mandate announcement. The "policy effect (%)" equals the estimate for the corresponding period divided by the baseline outcome level. For brevity purposes, the table only presents estimates for event times 0 and 2. Estimates for other periods are available upon request.

*** $p<0.01$, ** $p<0.05$, * $p<0.1$

© 2024 Wang Y et al. *JAMA Network Open*.

3.3 Robustness Check for Mandate Scope vs Mandate Stringency

3.3.1 Results for States with Broad Mandate Scope

eFigure 9. Event Study of Association Between State COVID-19 Vaccine Mandates and Proportion of HCWs Ever Vaccinated against COVID-19, States with Broad Mandate Scope

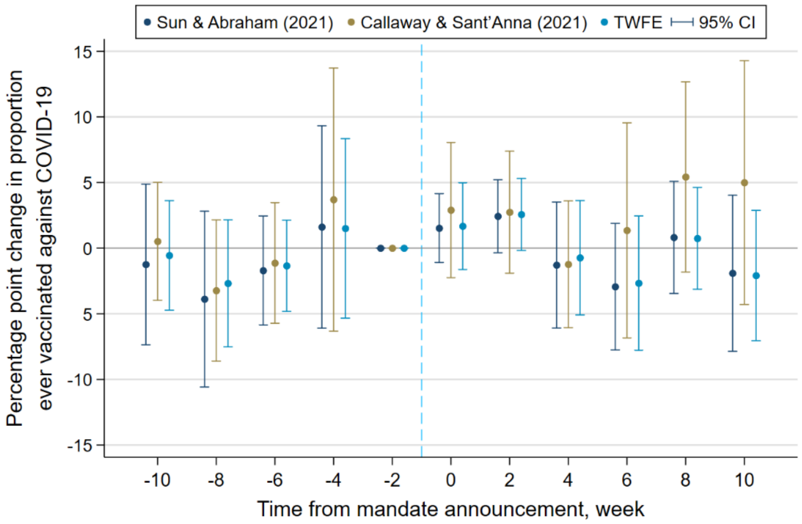

Note. Estimates with 95% CIs were derived from 3 difference-in-differences methods mentioned above using the biweekly HPS data with survey weights applied. Estimates here were adjusted for covariates described in the main text.

eFigure 10. Event Study of Association Between State COVID-19 Vaccine Mandates and Proportion of HCWs Who Completed or Intended to Complete the Primary Series, States with Broad Mandate Scope

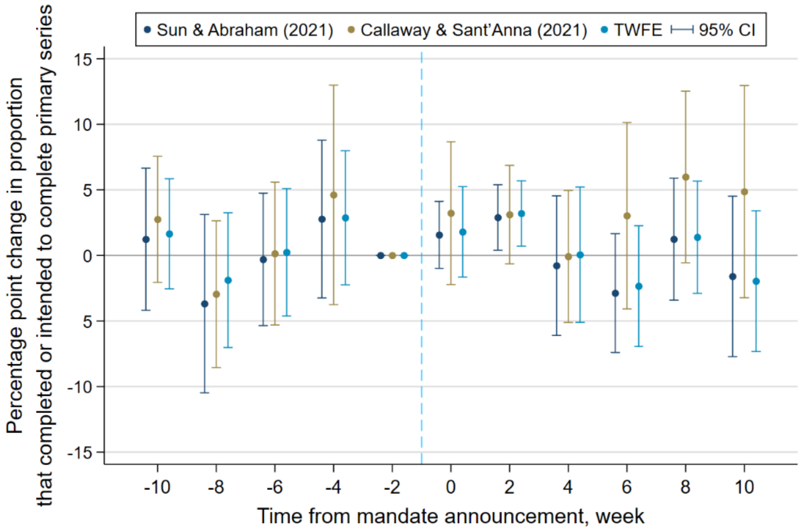

Note. Estimates with 95% CIs were derived from 3 difference-in-differences methods mentioned above using the biweekly HPS data with survey weights applied. Estimates here were adjusted for covariates described in the main text.

© 2024 Wang Y et al. *JAMA Network Open*.

eTable 6. Estimates of Associations Between State COVID-19 Vaccine Mandates and Vaccine Uptake Among HCWs, States with Broad Mandate Scope

	Outcome 1			Outcome 2		
	**S&A**	**C&S**	**TWFE**	**S&A**	**C&S**	**TWFE**
Event time 0	1.53	2.90	1.67	1.57	3.22	1.79
	[-1.18, 4.23]	[-2.25, 8.05]	[-1.74, 5.08]	[-1.07, 4.20]	[-2.22, 8.67]	[-1.77, 5.35]
	(0.26)	(0.27)	(0.33)	(0.24)	(0.25)	(0.31)
Event time 2	2.43*	2.74	2.57*	2.89**	3.11	3.20**
	[-0.44, 5.30]	[-1.91, 7.40]	[-0.26, 5.40]	[0.31, 5.47]	[-0.64, 6.87]	[0.63, 5.77]
	(0.09)	(0.25)	(0.07)	(0.03)	(0.10)	(0.02)
Baseline outcome level (%)		87.8			85.63	
Policy effect at time 0 (%)	1.74	3.30	1.91	1.83	3.77	2.10
Policy effect at time 2 (%)	2.77	3.12	2.93	3.38	3.63	3.74
N			27 216			

Note. Estimates were based on biweekly data and were adjusted for covariates. The "outcome 1" corresponds to proportion of HCWs ever vaccinated against COVID-19; the "outcome 2" corresponds to proportion of HCWs who completed or intended to complete the primary series. The 95% confidence interval (CI) in square brackets; the *P* value in parentheses. The "baseline outcome level" is the weighted mean of the outcome variable in the period immediately before mandate announcement. The "policy effect (%)" equals the estimate for the corresponding period divided by the baseline outcome level. For brevity purposes, the table only presents estimates for event times 0 and 2. Estimates for other periods are available upon request.

** $p<0.05$, * $p<0.1$

© 2024 Wang Y et al. *JAMA Network Open*.

3.3.2 Results for States with Broad Mandate Scope and a Test-out Option

eFigure 11. Event Study of Association Between State COVID-19 Vaccine Mandates and Proportion of HCWs Ever Vaccinated against COVID-19, States with Broad Mandate Scope and a Test-out Option

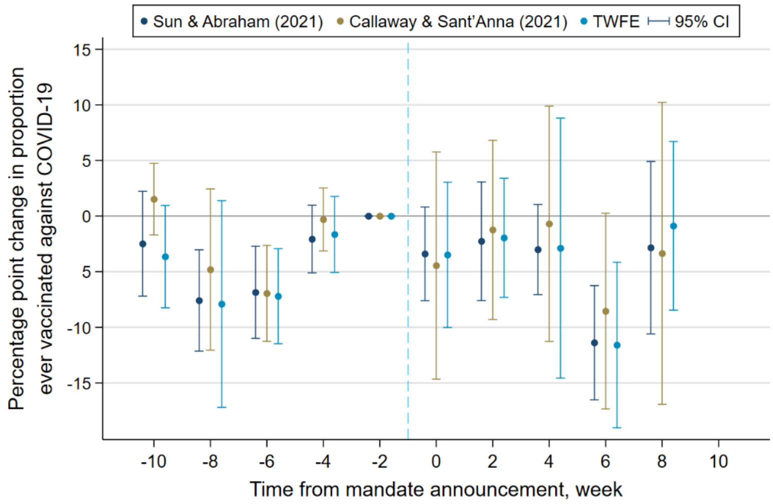

Note. Estimates with 95% CIs were derived from 3 difference-in-differences methods mentioned above using the biweekly HPS data with survey weights applied. Estimates here were adjusted for covariates. The post-mandate period of this subgroup did not extend to event time 10.

© 2024 Wang Y et al. *JAMA Network Open*.

eFigure 12. Event Study of Association Between State COVID-19 Vaccine Mandates and Proportion of HCWs Who Completed or Intended to Complete the Primary Series, States with Broad Mandate Scope and a Test-out Option

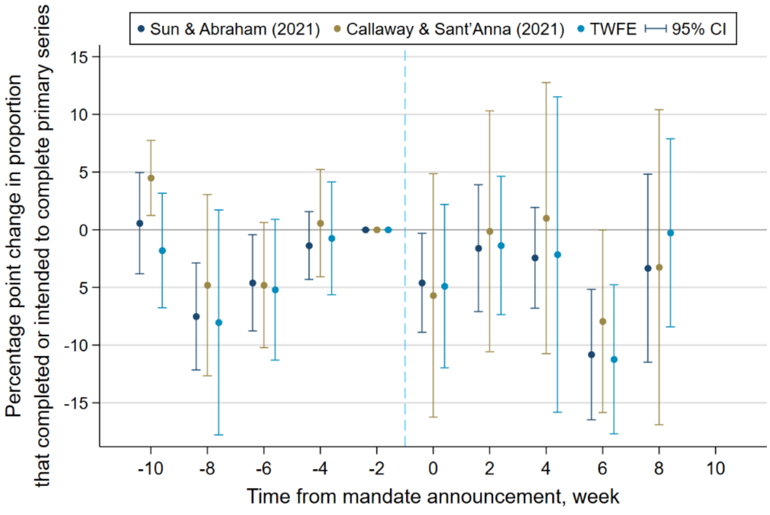

Note. Estimates with 95% CIs were derived from 3 difference-in-differences methods mentioned above using the biweekly HPS data with survey weights applied. Estimates here were adjusted for covariates. The post-mandate period of this subgroup did not extend to event time 10.

© 2024 Wang Y et al. *JAMA Network Open*.

eTable 7. Estimates of Associations Between State COVID-19 Vaccine Mandates and Vaccine Uptake Among HCWs, States with Broad Mandate Scope and a Test-out Option

	Outcome 1			Outcome 2		
	**S&A**	**C&S**	**TWFE**	**S&A**	**C&S**	**TWFE**
Event time 0	-3.39	-4.45	-3.49	-4.60**	-5.70	-4.89
	[-7.78, 0.99]	[-14.66, 5.76]	[-10.28, 3.31]	[-9.06, -0.14]	[-16.25, 4.85]	[-12.25, 2.47]
	(0.12)	(0.39)	(0.30)	(0.04)	(0.29)	(0.19)
Event time 2	-2.26	-1.24	-1.95	-1.60	-0.13	-1.37
	[-7.81, 3.29]	[-9.29, 6.81]	[-7.52, 3.61]	[-7.33, 4.12]	[-10.57, 10.30]	[-7.60, 4.87]
	(0.41)	(0.76)	(0.48)	(0.57)	(0.98)	(0.66)
Baseline outcome level (%)	93.10			91.97		
N	20 397					

Note. Estimates were based on biweekly data and were adjusted for covariates. The "outcome 1" corresponds to proportion of HCWs ever vaccinated against COVID-19; the "outcome 2" corresponds to proportion of HCWs who completed or intended to complete the primary series. The 95% confidence interval (CI) in square brackets; the *P* value in parentheses. The "baseline outcome level" is the weighted mean of the outcome variable in the period immediately before mandate announcement. For brevity purposes, the table only presents estimates for event times 0 and 2. Estimates for other periods are available upon request.

** $p<0.05$

© 2024 Wang Y et al. *JAMA Network Open*.

3.3.3 Results for States with Broad Mandate Scope and No Test-out Option

eFigure 13. Event Study of Association Between State COVID-19 Vaccine Mandates and Proportion of HCWs Ever Vaccinated against COVID-19, States with Broad Mandate Scope and No Test-out Option

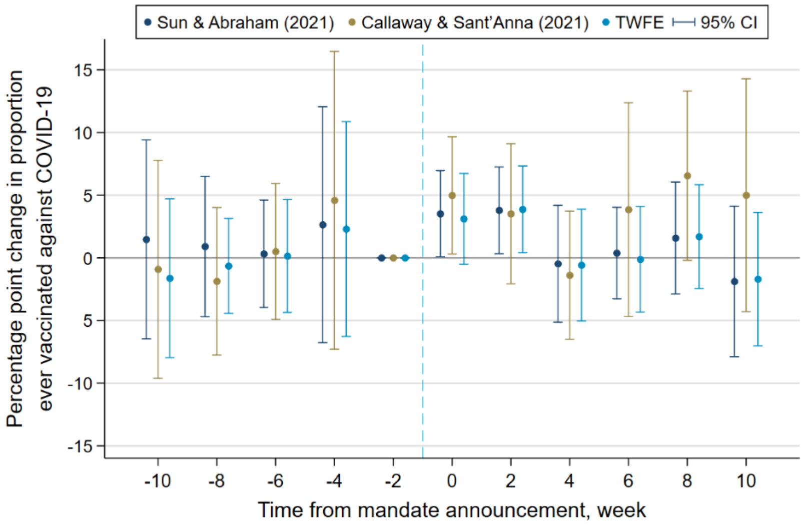

Note. Estimates with 95% CIs were derived from 3 difference-in-differences methods mentioned above using the biweekly HPS data with survey weights applied. Estimates here were adjusted for covariates described in the main text.

eFigure 14. Event Study of Association Between State COVID-19 Vaccine Mandates and Proportion of HCWs Who Completed or Intended to Complete the Primary Series, States with Broad Mandate Scope and No Test-out Option

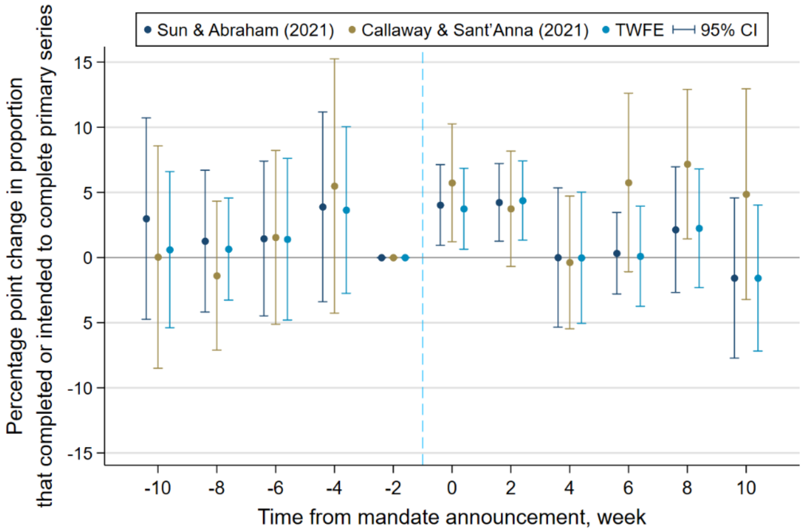

Note. Estimates with 95% CIs were derived from 3 difference-in-differences methods mentioned above using the biweekly HPS data with survey weights applied. Estimates here were adjusted for covariates described in the main text.

© 2024 Wang Y et al. *JAMA Network Open*.

eTable 8. Estimates of Associations Between State COVID-19 Vaccine Mandates and Vaccine Uptake Among HCWs, States with Broad Mandate Scope and No Test-out Option

	Outcome 1			Outcome 2		
	**S&A**	**C&S**	**TWFE**	**S&A**	**C&S**	**TWFE**
Event time 0	3.52*	4.98**	3.11	4.03**	5.74**	3.74**
	[-0.04, 7.08]	[0.31, 9.66]	[-0.63, 6.86]	[0.83, 7.24]	[1.22, 10.26]	[0.53, 6.96]
	(0.05)	(0.04)	(0.10)	(0.02)	(0.01)	(0.02)
Event time 2	3.79**	3.52	3.87**	4.23***	3.75*	4.38***
	[0.21, 7.38]	[-2.07, 9.11]	[0.29, 7.45]	[1.15, 7.32]	[-0.68, 8.17]	[1.23, 7.52]
	(0.04)	(0.22)	(0.03)	(0.01)	(0.10)	(0.01)
Baseline outcome level (%)		86.73			84.35	
Policy effect at time 0 (%)	4.06	5.75	3.59	4.78	6.80	4.44
Policy effect at time 2 (%)	4.37	4.06	4.47	5.02	4.44	5.19
N				25 530		

Note. Estimates were based on biweekly data and were adjusted for covariates. The "outcome 1" corresponds to proportion of HCWs ever vaccinated against COVID-19; the "outcome 2" corresponds to proportion of HCWs who completed or intended to complete the primary series. The 95% confidence interval (CI) in square brackets; the *P* value in parentheses. The "baseline outcome level" is the weighted mean of the outcome variable in the period immediately before mandate announcement. The "policy effect (%)" equals the estimate for the corresponding period divided by the baseline outcome level. For brevity purposes, the table only presents estimates for event times 0 and 2. Estimates for other periods are available upon request.

*** p<0.01, ** p<0.05, * p<0.1

3.4 Stratified Analysis by Ages of HCWs

3.4.1 Results for HCWs Aged 25-49

eFigure 15. Event Study of Association Between State COVID-19 Vaccine Mandates and Proportion of HCWs Ever Vaccinated against COVID-19, HCWs Aged 25-49

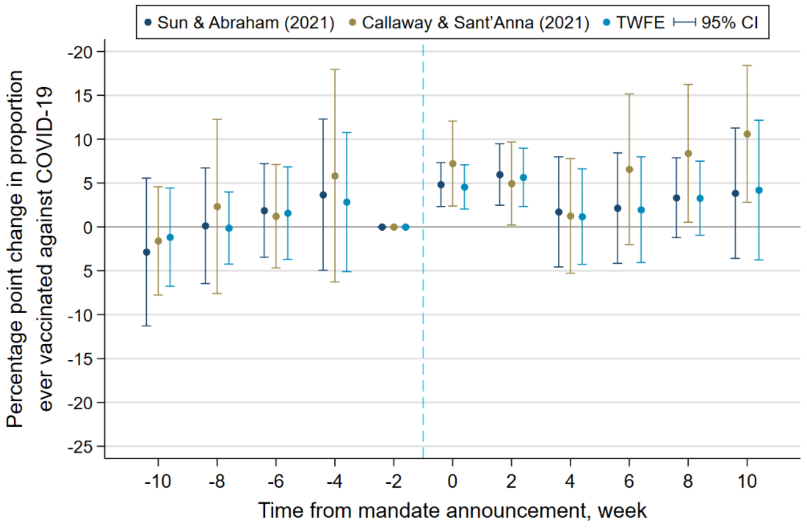

Note. Estimates with 95% CIs were derived from 3 difference-in-differences methods mentioned above using the biweekly HPS data with survey weights applied. Estimates here were adjusted for covariates described in the main text.

eFigure 16. Event Study of Association Between State COVID-19 Vaccine Mandates and Proportion of HCWs Who Completed or Intended to Complete the Primary Series, HCWs Aged 25-49

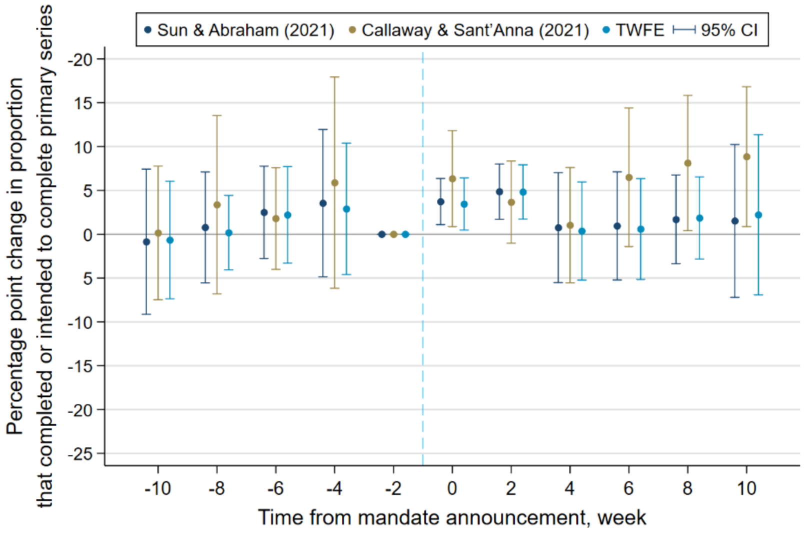

Note. Estimates with 95% CIs were derived from 3 difference-in-differences methods mentioned above using the biweekly HPS data with survey weights applied. Estimates here were adjusted for covariates described in the main text.

© 2024 Wang Y et al. *JAMA Network Open*.

eTable 9. Estimates of Associations Between State COVID-19 Vaccine Mandates and Vaccine Uptake Among HCWs Aged 25-49

	Outcome 1			Outcome 2		
	**S&A**	**C&S**	**TWFE**	**S&A**	**C&S**	**TWFE**
Event time 0	4.84***	7.23***	4.56***	3.72***	6.34**	3.45**
	[2.26, 7.41]	[2.39, 12.07]	[1.96, 7.15]	[1.02, 6.43]	[0.86, 11.82]	[0.39, 6.51]
	(< 0.001)	(0.003)	(0.001)	(0.01)	(0.02)	(0.03)
Event time 2	5.97***	4.95**	5.66***	4.86***	3.66	4.82***
	[2.37, 9.57]	[0.22, 9.69]	[2.24, 9.07]	[1.61, 8.10]	[-1.03, 8.35]	[1.64, 8.00]
	(0.002)	(0.04)	(0.002)	(0.004)	(0.13)	(0.004)
Baseline outcome level (%)		84.26			83.98	
Policy effect at time 0 (%)	5.74	8.58	5.41	4.43	7.55	4.10
Policy effect at time 2 (%)	7.09	5.88	6.71	5.78	4.36	5.74
N			19 247			

Note. Estimates were based on biweekly data and were adjusted for covariates. The "outcome 1" corresponds to proportion of HCWs ever vaccinated against COVID-19; the "outcome 2" corresponds to proportion of HCWs who completed or intended to complete the primary series. The 95% confidence interval (CI) in square brackets; the *P* value in parentheses. The "baseline outcome level" is the weighted mean of the outcome variable in the period immediately before mandate announcement. The "policy effect (%)" equals the estimate for the corresponding period divided by the baseline outcome level. For brevity purposes, the table only presents estimates for event times 0 and 2. Estimates for other periods are available upon request.

*** $p<0.01$, ** $p<0.05$

© 2024 Wang Y et al. *JAMA Network Open*.

3.4.2 Results for HCWs Aged 50-64

eFigure 17. Event Study of Association Between State COVID-19 Vaccine Mandates and Proportion of HCWs Ever Vaccinated against COVID-19, HCWs Aged 50-64

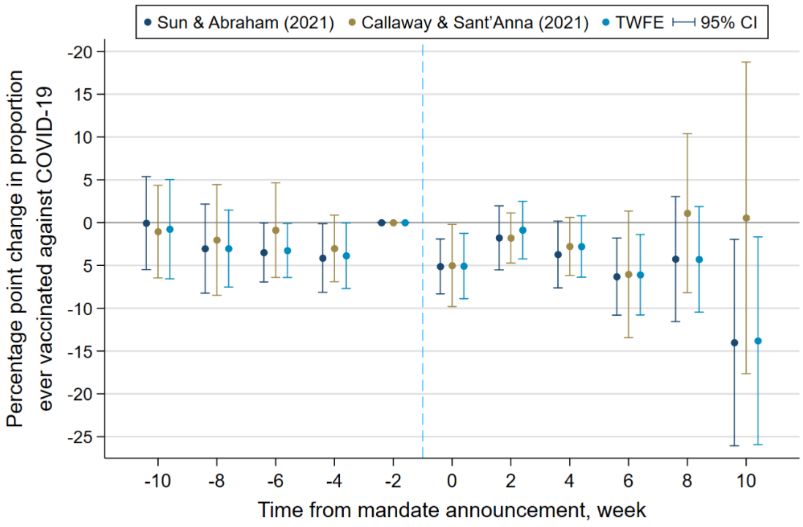

Note. Estimates with 95% CIs were derived from 3 difference-in-differences methods mentioned above using the biweekly HPS data with survey weights applied. Estimates here were adjusted for covariates described in the main text.

© 2024 Wang Y et al. *JAMA Network Open*.

eFigure 18. Event Study of Association Between State COVID-19 Vaccine Mandates and Proportion of HCWs Who Completed or Intended to Complete the Primary Series, HCWs Aged 50-64

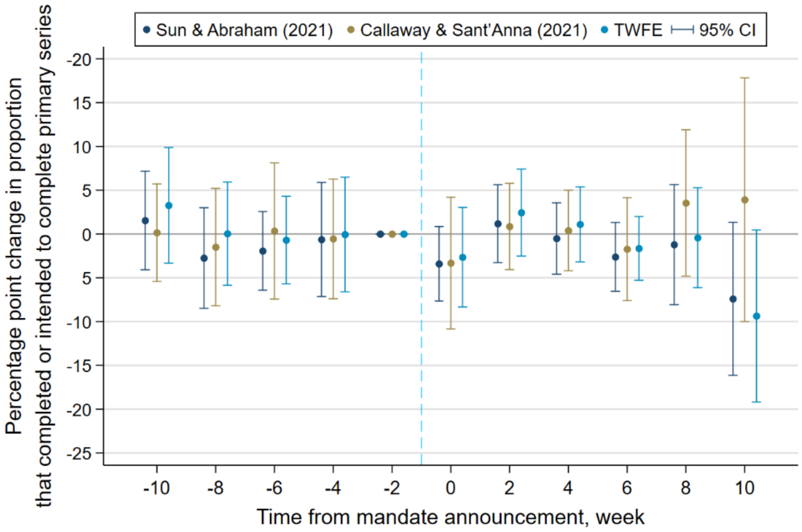

Note. Estimates with 95% CIs were derived from 3 difference-in-differences methods mentioned above using the biweekly HPS data with survey weights applied. Estimates here were adjusted for covariates described in the main text.

© 2024 Wang Y et al. *JAMA Network Open*.

eTable 10. Estimates of Associations Between State COVID-19 Vaccine Mandates and Vaccine Uptake Among HCWs Aged 50-64

	Outcome 1			Outcome 2		
	**S&A**	**C&S**	**TWFE**	**S&A**	**C&S**	**TWFE**
Event time 0	-5.12***	-5.02**	-5.07**	-3.4	-3.32	-2.65
	[-8.42, -1.82]	[-9.82, -0.22]	[-9.00, -1.14]	[-7.78, 0.97]	[-10.84, 4.20]	[-8.50, 3.19]
	(0.003)	(0.04)	(0.01)	(0.12)	(0.39)	(0.37)
Event time 2	-1.78	-1.80	-0.87	1.18	0.86	2.44
	[-5.62, 2.06]	[-4.72, 1.13]	[-4.33, 2.59]	[-3.39, 5.76]	[-4.07, 5.78]	[-2.66, 7.54]
	(0.36)	(0.23)	(0.62)	(0.61)	(0.73)	(0.34)
Baseline outcome level (%)		95.94			90.69	
N	11 895					

Note. Estimates were based on biweekly data and were adjusted for covariates. The "outcome 1" corresponds to proportion of HCWs ever vaccinated against COVID-19; the "outcome 2" corresponds to proportion of HCWs who completed or intended to complete the primary series. The 95% confidence interval (CI) in square brackets; the *P* value in parentheses. The "baseline outcome level" is the weighted mean of the outcome variable in the period immediately before mandate announcement. For brevity purposes, the table only presents estimates for event times 0 and 2. Estimates for other periods are available upon request.

*** $p<0.01$, ** $p<0.05$

© 2024 Wang Y et al. *JAMA Network Open*.

eReferences

1. Vaccine Mandates and Prohibitions. Thomson Reuters Practical Law. Accessed November 10, 2022, https://content.next.westlaw.com/practical-law/document/I6c41215f69d111eaadfea82903531a62/COVID-19-Employment-Law-and-Development-Tracker?transitionType=Default&contextData=%28sc.Default%29#co_anchor_a589335
2. Emergency proclamation related to the COVID-19 response. Office of the Governor David Ige; August 5, 2021, Accessed December 5, 2022. https://dod.hawaii.gov/hiema/files/2021/08/2108026-ATG_Emergency-Proc-for-COVID-19-Response-distribution-signed.pdf
3. Governor Walz Announces Vaccination Requirements for State Agency Employees. Office of Governor Tim Walz; August 11, 2021, Accessed December 5, 2022. https://mn.gov/governor/covid-19/news/#/detail/appId/1/id/493652
4. Governor Sisolak signs emergency regulation related to COVID-19 vaccine. Officee of Governor Steve Sisolak; September 14, 2021 Accessed December 5, 2022. https://nvhealthresponse.nv.gov/wp-content/uploads/2021/09/Governor-Sisolak-signs-emergency-regulation-related-to-COVID-19-vaccine-.pdf
5. CORRECTION: Gov. Beshear Announces New Testing and Vaccine Program in State-Run Health Care Facilities. Office of the Governor Andy Beshear; August 2, 2021, Accessed December 5, 2022. https://www.kentucky.gov/Pages/Activity-stream.aspx?n=GovernorBeshear&prId=900
6. Dawson J. Idaho's lieutenant governor banned vaccine mandates while Gov. Little was out of town. National Public Radio. Updated October 6, 2021. Accessed December 4, 2022, https://www.npr.org/2021/10/06/1043788284/idahos-lieutenant-governor-banned-vaccine-mandates-while-gov-little-was-out-of-t
7. Nursing Home Employee Vaccination. Mississippi State Department of Health; June 14, 2021, Accessed December 5, 2022. https://msdh.ms.gov/msdhsite/_static/resources/14517.pdf
8. California Implements First-in-the-Nation Measures to Encourage State Employees and Health Care Workers to Get Vaccinated. Office of Governor Gavin Newsom; July 26, 2021, Accessed December 1, 2022. https://www.gov.ca.gov/2021/07/26/california-implements-first-in-the-nation-measures-to-encourage-state-employees-and-health-care-workers-to-get-vaccinated/
9. Order of the State Public Health Officer Health Care Worker Vaccine Requirement. California Department of Public Health. Updated March 3, 2023. Accessed March 15, 2023, https://www.cdph.ca.gov/Programs/CID/DCDC/Pages/COVID-19/Order-of-the-State-Public-Health-Officer-Health-Care-Worker-Vaccine-Requirement.aspx
10. Governor Polis Urges State Board of Health to Engage in Expedited Rulemaking to Require COVID-19 Vaccine for All Personnel Working Directly with Colorado’s Vulnerable Populations & Settings Where Coloradans Receive Essential Medical Care. Colorado Governor Jared Polis; August 17, 2021, Accessed December 2, 2022. https://www.colorado.gov/governor/news/6036-governor-polis-urges-state-board-health-engage-expedited-rulemaking-require-covid-19

© 2024 Wang Y et al. *JAMA Network Open*.

11. Governor Lamont Orders Long-Term Care Facility Staff in Connecticut To Be Vaccinated Against COVID-19. The Office of Governor Ned Lamont; August 6, 2021, Accessed December 2, 2022. https://portal.ct.gov/Office-of-the-Governor/News/Press-Releases/2021/08-2021/Governor-Lamont-Orders-Long-Term-Care-Facility-Staff-in-Connecticut-To-Be-Vaccinated
12. COVID-19 Vaccination and Testing Updates for Long-Term Care Facilities, Health Care Facilities, and State Employees. Governor John Carney; August 12, 2021, Accessed December 2, 2022. https://news.delaware.gov/2021/08/12/covid-19-vaccination-and-testing-updates-for-long-term-care-facilities-health-care-facilities-and-state-employees/
13. Mayor Bowser and DC Health Announce Vaccination Requirement for District Health Professionals. Government of The District of Columbia; August 16, 2021, Accessed December 2, 2022. https://mayor.dc.gov/release/mayor-bowser-and-dc-health-announce-vaccination-requirement-district-health-professionals
14. Governor Pritzker Announces COVID-19 Vaccine Requirement for Healthcare Workers, Pre-K-12 Teachers and Staff, Higher Education Personnel and Students to Slow Spread of Delta Variant. Office of Governor JB Pritzker; August 26, 2021, Accessed December 2, 2022. https://www.illinois.gov/news/press-release.23808.html#:~:text=Teachers%20and%20staff%20at%20pre,vaccination%20by%20September%205%2C%202021.
15. Mills Administration Requires Health Care Workers To Be Fully Vaccinated Against COVID-19 By October 1. Office of Governor Janet T. Mills; August 12, 2021, Accessed December 3, 2022. https://www.maine.gov/governor/mills/news/mills-administration-requires-health-care-workers-be-fully-vaccinated-against-covid-19-october
16. Governor Hogan Announces New Vaccination Protocols for Maryland Nursing Home and Hospital Workers. The Office of Governor Larry Hogan; August 18, 2021, Accessed December 3, 2022. https://governor.maryland.gov/2021/08/18/governor-hogan-announces-new-vaccination-protocols-for-maryland-nursing-home-and-hospital-workers/
17. Order of the Commissioner of Public Health: COVID-19 Public Health Emergency Order No. 2021-4. State Library of Massachusetts. Updated August 5, 2021. Accessed December 3, 2021, https://archives.lib.state.ma.us/handle/2452/847341
18. Governor Murphy Announces Vaccine Requirement for Workers in Health Care Facilities and High-Risk Congregate Settings. August 2, 2021, Accessed December 3, 2022. https://nj.gov/governor/news/news/562021/approved/20210802a.shtml
19. Public Health Order Requiring All School Workers Comply with Certain Health Requirements and Requiring Congregate Care Facility Workers, Hospital Workers, and Employees of the Office of the Governor be Fully Vaccinated. New Mexico Department of Health; August 17, 2021, Accessed December 4, 2022. https://cv.nmhealth.org/wp-content/uploads/2021/08/081721-PHO-Vaccines.pdf
20. Governor Cuomo Announces Patient-Facing Healthcare Workers at State-Run Hospitals Will Be Required to Get Vaccinated for COVID-19 by Labor Day. Office of Governor Andrew M. Cuomo; July 28, 2021, Accessed December 2, 2022.

© 2024 Wang Y et al. *JAMA Network Open.*

https://www.governor.ny.gov/news/governor-cuomo-announces-patient-facing-healthcare-workers-state-run-hospitals-will-be

21. Governor Cuomo Announces COVID-19 Vaccination Mandate for Healthcare Workers. Office of Governor Andrew M. Cuomo; August 16, 2021, Accessed December 2, 2022. https://www.governor.ny.gov/news/governor-cuomo-announces-covid-19-vaccination-mandate-healthcare-workers
22. Governor Kate Brown Announces New Health and Safety Rule for Employees in Health Care Settings to Help Prevent Delta Variant Spread. Office of the Governor Kate Brown; August 4, 2021, Accessed December 3, 2021. https://mailchi.mp/oregon/news-releasegovernor-kate-brown-announces-new-health-and-safety-rule-for-employees-in-health-care-settings-to-help-prevent-delta-variant-spread?e=9cb5cfb484
23. COVID-19 Updates and Information. The State of Oregon. Updated December 13, 2021. Accessed December 3, 2022, https://www.oregon.gov/otlb/Pages/COVID-19-Updates-and-Information.aspx#gsc.tab=0
24. Governor Wolf Announces ‘Vaccine Or Test’ Requirement For Commonwealth Employees In Health Care And High-Risk Congregate Facilities. Pennsylvania Pressroom; August 10, 2021, Accessed December 3, 2022. https://www.media.pa.gov/Pages/military-and-veteran-affairs-details.aspx?newsid=501
25. State to Require Immunization Against COVID-19 for All Licensed Healthcare Workers. Rhode Island Department of Health; August 18, 2021, Accessed December 3, 2022. https://www.ri.gov/press/view/41888
26. Governor, RIDOH Announce Enforcement Strategy for October 1 Healthcare Worker and Healthcare Facility COVID-19 Vaccination Requirements. Office of the Governor Dan McKee; September 21, 2021, Accessed December 4, 2022.
27. Inslee issues proclamation requiring vaccination for most state employees, health and long-term care workers. Office of Governor Jay Inslee; August 9, 2021, Accessed December 4, 2022. https://governor.wa.gov/news/2021/inslee-issues-proclamation-requiring-vaccination-most-state-employees-health-and-long-term-care
28. Sun L, Abraham S. Estimating dynamic treatment effects in event studies with heterogeneous treatment effects. *Journal of Econometrics*. 2021;225(2):175-199. doi:10.1016/j.jeconom.2020.09.006
29. Callaway B, Sant’Anna PHC. Difference-in-Differences with multiple time periods. *Journal of Econometrics*. 2021;225(2):200-230. doi:10.1016/j.jeconom.2020.12.001

© 2024 Wang Y et al. *JAMA Network Open*.
