# Supplementary material for: State COVID-19 Vaccine Mandates and Uptake Among Health Care Workers in the US
Source: JAMA Netw Open. 2024 Aug 14;7(8):e2426847. doi: 10.1001/jamanetworkopen.2024.26847 (PMC11325213; doi:10.1001/jamanetworkopen.2024.26847)
Supplement: Supplement 2. — Data Sharing Statement [file jamanetwopen-e2426847-s002.pdf]

Data Sharing Statement

Wang. State COVID-19 Vaccine Mandates and Uptake Among Health Care Workers in the US. *JAMA Netw Open*. Published August 14, 2024. doi:10.1001/jamanetworkopen.2024.26847

Data
Data available: Yes
Data types: Deidentified participant data
How to access data: The Household Pulse Survey from the US Census Bureau: https://www.census.gov/programs-surveys/household-pulse-survey.html
When available: With publication

Supporting Documents
Document types: Statistical/analytic code
How to access documents: Statistical/analytic code is available upon request. Email: cfstoecker@tulane.edu
When available: With publication

Additional Information
Who can access the data: anyone requesting the data
Types of analyses: for any purpose
Mechanisms of data availability: without investigator support
